# Effects of transcranial direct current stimulation using a commercially available device on gait in Parkinson’s disease with freezing of gait

**DOI:** 10.1371/journal.pone.0330286

**Published:** 2025-08-21

**Authors:** Atsushi Umemura, Kazunori Sato, Hirokazu Iwamuro, Saki Uchiyama, Naotake Yanagisawa, Akihide Kondo, Nobutaka Hattori

**Affiliations:** 1 Department of Neurosurgery, Juntendo University Graduate School of Medicine, Tokyo, Japan; 2 Department of Research and Therapeutics for Movement Disorders, Juntendo University Graduate School of Medicine, Tokyo, Japan; 3 Department of Rehabilitation Medicine, Juntendo University Hospital, Tokyo, Japan; 4 Medical Technology Innovation Center, Clinical Research and Trial Center, Juntendo University, Tokyo, Japan; 5 Department of Neurology, Juntendo University Graduate School of Medicine, Tokyo, Japan; University of Minho: Universidade do Minho, PORTUGAL

## Abstract

**Background:**

Gait disturbance is one of the most disabling symptoms in patients with Parkinson’s disease (PD). In particular, freezing of gait (FOG) is a major cause of falls and significantly impairs the activities of daily living. Neither drug therapy nor current neuromodulation therapy are effective against FOG in most patients, so the development of new treatments is needed.

**Materials and Methods:**

This pilot study investigated the effects and safety of neuromodulation by single session anodal transcranial direct current stimulation (tDCS) to the cerebral cortex (supplementary motor area [SMA] or primary motor cortex [M1]) on gait in PD patients with FOG. We purposely used a commercially available tDCS device (Halo Sport 2; Halo Neuroscience, San Francisco, CA, USA) that patients can use themselves. This single arm, open label, sham-controlled study enrolled 20 advanced PD patients who complained of FOG.

**Results:**

Analysis of the 30-second walking distance, which was the primary endpoint, and the 10-m natural walking and 360-degree rotation tests showed that neither SMA nor M1 stimulation had a superior effect compared to sham stimulation. On the other hand, only SMA stimulation showed a significant improvement in the required time and the number of steps in the Timed Up and Go test compared to sham stimulation (*p* < 0.05).

**Conclusions:**

These results suggest that tDCS to the SMA using a commercially available device may improve the actual walking ability of PD patients with FOG.

## 1. Introduction

Parkinson’s disease (PD) is a progressive nervous disorder caused by degeneration of dopamine-producing cells in the substantia nigra. The main symptoms are movement-related, including tremor, rigidity, bradykinesia, postural instability, gait disturbance, and others. The underlying pathophysiology involves complex alterations in the dopaminergic system, particularly in the basal ganglia circuitry. Although no radical treatment for PD has been established, replacement therapy with dopaminergic medication and deep brain stimulation (DBS) of the subthalamic nucleus and the globus pallidus internus is effective to correct functional abnormalities in the basal ganglia. Most of the motor symptoms are controllable through these treatments. However, some motor symptoms remain resistant to treatment. [1–4]

Gait disturbance is one of the most disabling symptoms in PD. In particular, freezing of gait (FOG) is a condition in which the sole of the foot becomes apparently stuck to the floor when starting to walk or changing direction, making it difficult to walk, and is a major cause of falls and significantly impairs the activities of daily living [5,6]. As for risk factors of FOG, gait disorders, Postural instability and gait difficulty (PIGD) phenotype, and lower striatal dopamine transporter (DAT) uptake were known as independent risk factors of FOG with consistent evidence [7]. The effects of drug therapy and DBS on FOG vary, and it is particularly difficult to treat FOG in a medication-on period [8–10]. Therefore, new treatments are desirable.

The pathophysiology of FOG is not yet clear, but decreased activity of the supplementary motor area (SMA) may be involved [11]. The SMA is located in front of the primary motor cortex (M1) and is involved in the planning, coordination, and execution of self-initiated (uncued) movements. Neuroimaging study with positron emission tomography demonstrated a deficit of self-initiated movements in PD is due to underactivation of the SMA [12]. In addition, some functional magnetic resonance imaging studies have demonstrated reduced neural activity in both the basal ganglia and the cerebral cortex such as the SMA and frontal lobe [13–15].

Based on these observations, non-invasive neuromodulation of the SMA by repetitive transcranial magnetic stimulation (rTMS) or transcranial direct current stimulation (tDCS) has been evaluated to treat FOG [16]. Stimulation sites have included the M1, SMA, dorsolateral prefrontal cortex (DLPFC), and various combinations. rTMS has been approved by the Food and Drug Administration as a medical device because the pinpoint effect on the cerebral cortex produces stable effects. Some studies have demonstrated rTMS of the SMA rather than the M1 improved FOG in PD [16–18].

tDCS is a non-invasive stimulation method in which a weak direct current is passed through the skull from electrodes placed on the scalp, which modifies the cortical excitability directly below the electrode [19,20]. Under the anode, the cell membrane depolarizes and cortical excitability increases, whereas under the cathode, hyperpolarization occurs and excitability decreases. The effect of tDCS persists for a certain period of time (several minutes to an hour) even after turning off the stimulation, and tDCS may also change the synaptic plasticity [20,21]. However, tDCS has not been approved as a medical device because of the effects over a wide area of cerebral cortex and unstable effects. Compared to rTMS, tDCS devices are relatively inexpensive, small, and some are wearable, and various devices on the market can be used at home [22]. tDCS is a generally safe procedure when performed within standardized protocols. Therefore, tDCS can be easily used in rehabilitation settings. Several studies have examined the effects of tDCS on gait in PD patients [23]. Although the results have been inconsistent, some studies showed the effectiveness of M1, SMA, or DLPFC stimulation on FOG in PD [24.^25^, ^26^], with improvement even using a single-session approach [27]. On the other hand, there is also a report that tDCS to the SMA is ineffective against FOG [28].

This pilot study investigated the effects and safety of neuromodulation by single session anodal tDCS to the cerebral cortex (SMA or M1) on gait in PD patients with FOG. We purposely used a commercially available tDCS device that patients can use themselves.

## 2. Materials and methods

### 2.1. Participants

This was a single arm, open label, sham-controlled study. The study included patients who were admitted to the Department of Neurology or Neurosurgery at Juntendo University Hospital (Tokyo, Japan) for examination or treatment. Inclusion criteria were male and female patients aged 20 or older, patients who had been diagnosed with idiopathic PD according to the UK Brain Bank criteria, and patients who had symptoms of freezing of gait. Exclusion criteria were patients who use implantable medical or electronic devices such as deep brain stimulation, pacemakers and defibrillators, patients who have other neuropsychological disorder, history of epilepsy or seizures, injuries or defects in the stimulated part of the skull, and a metal coil in the skull.

**Fig 1:**
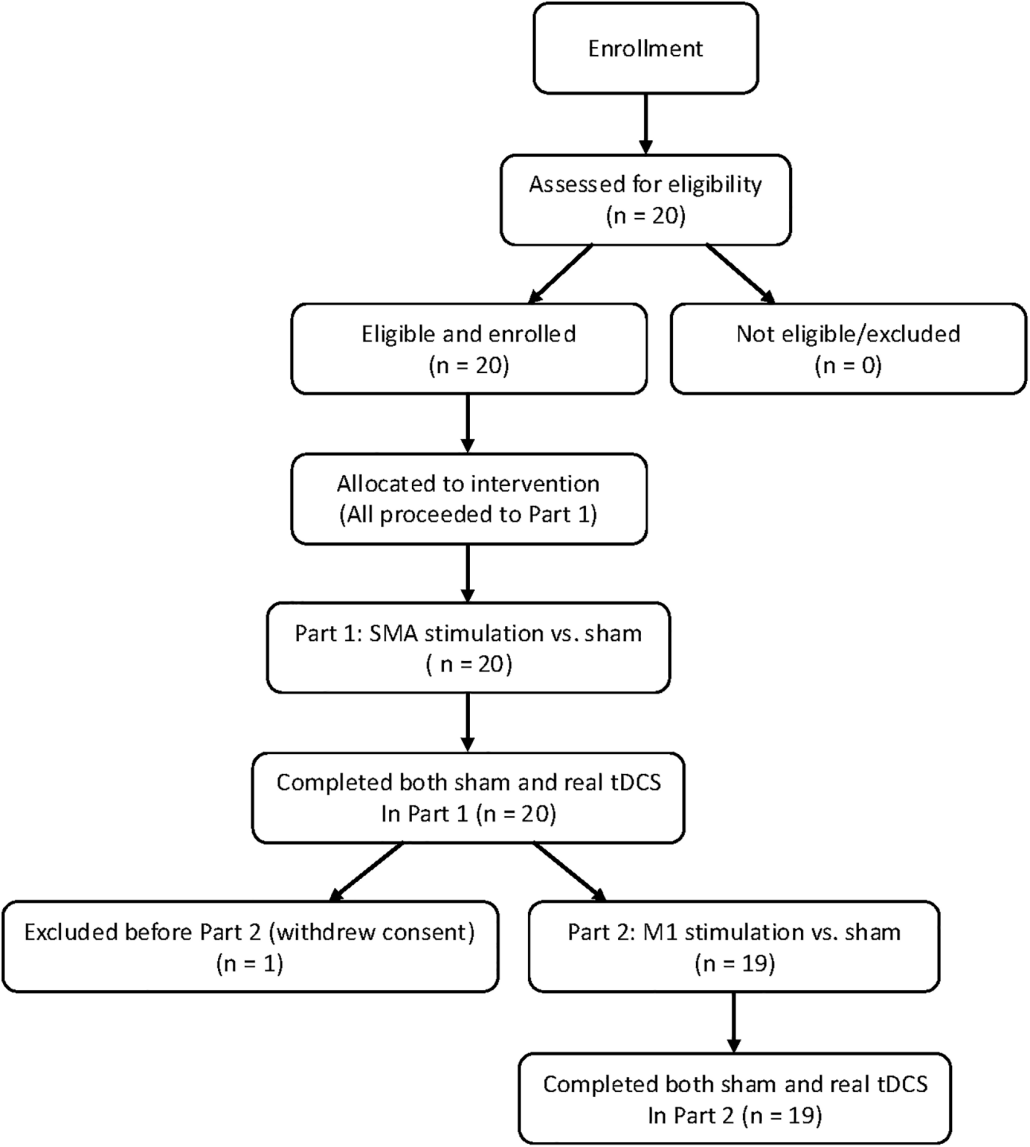
CONSORT participant flow diagram. A total of 20 participants were enrolled. All participants completed Part 1 (SMA vs. sham), and 19 completed Part 2 (M1 vs. sham). One participant withdrew after Part 1.

We recruited 20 patients between January 1, 2021 and December 31, 2022 (Fig. 1). Because this is an exploratory study, there is not enough information to calculate the target number of cases based on statistical power. Therefore, we estimated the number of patients who meet the selection criteria and who can consent to participate in the study from the annual number of patients hospitalized with the disease at our hospital, and set the target number at 20 cases, which was the number that can be implemented within the study period.

Upon registration in this study, the principal investigator shall provide the patient with a sufficient explanation of this study orally and in writing, and obtain written consent to participate in the study. Patient registration was conducted using Research Electronic Data Capture (REDCap) managed by Juntendo University.

After obtaining consent, eligibility was confirmed. Then, various data that show the characteristics of Parkinson’s disease in each patient were collected in the screening period. They were Japanese version of FOG questionnaire (FOG-Q) [29,30], Movement Disorder Society-Unified Parkinson’s Disease Rating Scale (MDS-UPDRS) part III [31] in the medication-off period and in the medication-on period, Japanese version of Mini-Mental State Examination (MMSE) [32,33], Japanese version of Frontal Assessment Battery (FAB) [34,35], Japanese version of Montreal Cognitive Assessment (MoCA) [36,37], and Japanese version of Parkinson’s Disease Questionnaire-39 (PDQ-39) [38,39].

The study was approved by our Institutional Review Board (approved number: J20-005), and registered to the Japan Registry of Clinical Trial (identifier number: jRCTs032200173).

### 2.2. tDCS device

**Fig 2:**
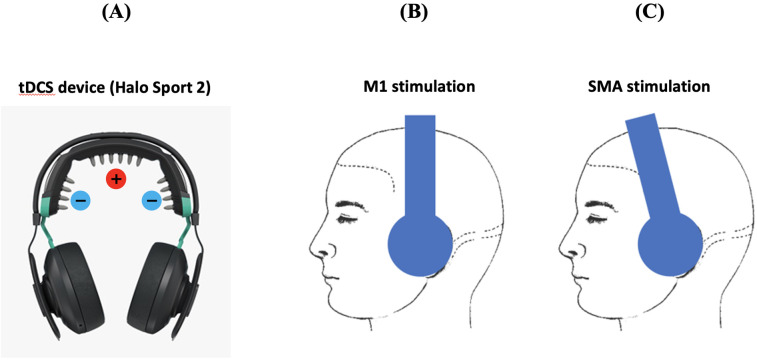
Headphone type wearable tDCS device **(A)**. Head placement for M1 stimulation (**B**) and SMA stimulation **(C).**

The commercially available tDCS device (Halo Sport 2; Halo Neuroscience, San Francisco, CA, USA) was originally developed to improve motor performance in athletes (Fig. 2A) [40–42]. This tDCS device appears similar to headphones and incorporates the anode on the vertex of the head (Cz) and two cathodes located at C5 and C6. The size of the electrodes affixed to the scalp was 28 cm^2^ (6.4 cm x 4.4 cm). Activation of the anode at the vertex (Cz) stimulates the M1 of the bilateral leg areas (Fig. 2B). The SMA is located on the midline surface of the hemisphere just anterior to the M1, so SMA activation can be achieved by relocating the anode to 2 cm anterior to the vertex (Fig. 2C). Stimulation trial used current of 2 mA and duration of 20 minutes. Sham stimulation trial required the device to be worn for 20 minutes with no current applied. We did not performed 30 seconds activation of tDCS as was done in previous studies.

### 2.3. Study design

**Fig 3:**
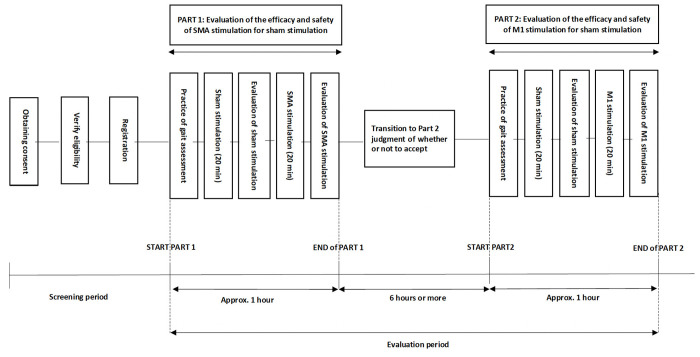
The study design.

This was a single arm, open label, sham-controlled study investigating the effect of tDCS on gait in patients with PD manifesting as FOG. Two tests were conducted in each patient. PART 1 evaluated the effectiveness and safety of SMA stimulation against sham stimulation, and PART 2 evaluated the effectiveness and safety of M1 stimulation against sham stimulation. Patients with significant motor fluctuation underwent evaluation in a medication-off period, in which FOG was more likely to occur. Besides, it was necessary to evaluate sham stimulation and real stimulation continuously in a short period of time in order to evaluate them under the similar conditions. The study design is shown in Fig. 3.

In PART 1, the tDCS device was attached to the head for 20 minutes while sitting in a chair and resting to achieve sham stimulation, then the device was removed and the first series of gait evaluations was performed immediately. The tDCS device was then attached again and SMA stimulation was given at 2 mA for 20 minutes in the same resting state, after which the device was removed and a second series of gait evaluations was performed immediately. Patients were not informed about the presence or absence of real stimulation. However, the patients may feel discomfort on their scalp during actual tDCS stimulation, so blinding the patient to the presence or absence of stimulation is difficult. Regarding the order of real and sham stimulation, the effect of tDCS persist for a while after stimulation has ended, so we had no choice but to perform sham stimulation first and then real stimulation. PART 1 evaluation took about 60 minutes to complete all procedures.

In PART 2, a tDCS device was attached and sham stimulation was performed for 20 minutes, followed by gait evaluation. The tDCS device was then attached again and M1 stimulation was performed for 20 minutes at 2 mA, followed by gait evaluation. PART 2 testing was conducted after a sufficient washout period of 48 hours to completely eliminate the effects of tDCS stimulation in PART 1 [21].

### 2.4. Assessment of walking ability

We indirectly assessed FOG by assessment of walking ability. Walking ability was assessed with several walking-related tests. The primary endpoint was walking distance in 30 seconds. This is a simple test that measures the walking distance in 30 seconds in a rehabilitation track, but the results are likely to vary greatly depending on the severity of FOG. Secondary endpoints were 10-m natural walking, Timed Up and Go (TUG) test, and 360-degree rotation test. In the 10-m natural walking test, the walking time and number of steps taken to walk 10 meters were measured. The TUG test evaluates the time and number of streps of a movement sequence that involves rising from a chair, walking three meters, turning, returning to the chair, and sitting down on the same chair at a comfortable pace [43]. The 360-degree rotation test measures the time and number of steps required to rotate 360 degrees clockwise or counterclockwise in a standing position. Some PD patients show significant FOG, especially when changing direction, so the 360-degree rotation test is useful for detecting FOG.

The basis for setting the primary endpoint is that patients with FOG adopt slower walking speeds, but may not be able to walk at all in severe cases. Even in such cases, walking distance as the primary endpoint can be retained as data. On the other hand, the 10-m walking time, which was set as a secondary endpoint, may not be available in such severe cases, so the patient may have to be excluded. Therefore, walking distance was set as the primary endpoint.

These assessment was performed by a physiotherapist (K.S.). Assessment was not blinded to the evaluator. But it doesn’t matter because the evaluation items are only walking distance, time, and number of steps, which can be evaluated completely objectively. These evaluations were performed in a spacious rehabilitation room. The actual walking conditions during these evaluations were recorded on video in all participants. All participants practiced the gait assessment without wearing the tDCS device separately from the actual test. This series of gait assessment can be performed repeatedly in healthy individuals, but it places a significant physical burden on advanced PD patients. Therefore, evaluation was performed once each after sham stimulation and after actual stimulation in the actual test, and these results were analyzed.

### 2.5. Statistical analysis

In this study, we used REDCap as a data management tool. After data were fixed, statistical analysis was performed by statisticians. This study is a one-arm study, meaning no group comparisons were performed because both sham and real stimulations were done on the same subjects, making only intra-subject comparisons. Therefore, the outcome measurements between sham and real stimulation in each group were compared by the Wilcoxon signed-rank sum test using SAS software version 9.4. Probability values of *p* < 0.05 were considered statistically significant. In patients with advanced PD, the baseline may vary significantly depending on the time of evaluation due to motor fluctuations. Therefore, we conducted the assessment using separate controls (sham) for Part 1 and Part 2, and we did not apply correction for multiplicity.

## 3. Result

### 3.1. Participant characteristics

This study enrolled 20 patients with advanced PD who complained of FOG (6 men and 16 women, age 64.8 ± 6.5 years, duration of disease 13.0 ± 4.2 years, mean ± standard deviation [SD]).

The mean ± SD score for the FOG-Q was 16.7 ± 4.2, MDS-UPDRS part III in the medication-off period was 40.6 ± 12.2, MDS-UPDRS III in the medication-on period was 14.4 ± 6.3, MMSE was 29.0 ± 1.4, FAB was 16.3 ± 1.5, MoCA was 26.7 ± 2.4, and PDQ-39 was 56.4 ± 29.2.

### 3.2. Effect of tDCS on gait

Twenty patients were enrolled in this study. However, one participant who completed PART 1 withdrew consent before proceeding to PART 2. Therefore, Part 1 included 20 participants and Part 2 included 19 participants. In addition, One participant was unable to obtain data on 360-degree rotation only in both Part 1 and Part 2.

All patients completed a gait assessment and could provide data on walking distance, time, and number of steps. However, the degree of FOG varied between patients at the time of evaluation. Some patients with severe FOG required considerable time for gait evaluation. The results of all walking evaluations are shown in Table 1.

**Table 1:** Result of walking evaluation with sham and tDCS stimulation.

		PART 1 (n = 20)			PART 2 (n = 19)	
	sham stim.	SMA stim	*p* value	sham stim.	M1 stim	*p* value
30-second walking distance (m)	23.2 ± 8.3	23.8 ± 8.3	0.49	24.0 ± 8.5	26.2 ± 9.7	0.23
10m natural walking						
time (sec)	16.2 ± 7.0	17.9 ± 19.3	0.26	15.2 ± 6.6	13.7 ± 5.9	0.12
number of steps	30.9 ± 15.4	35.6 ± 42.3	0.49	28.7 ± 1.6	26.8 ± 11.1	0.38
Timed Up and Go test						
time (sec)	30.4 ± 31.5	20.6 ± 12.5	0.03	21.3 ± 14.8	17.9 ± 8.7	0.49
number of steps	50.0 ± 41.6	35.9 ± 26.1	0.047	38.2 ± 28.2	32.9 ± 18.6	0.81
360 degree rotation test						
Clockwise rotation						
time (sec)	11.7 ± 11.8	11.5 ± 17.2	0.25	8.1 ± 3.6	9.3 ± 7.2	0.71
number of steps	22.0 ± 9.8	20.1 ± 10.8	0.43	21.2 ± 10.2	19.5 ± 8.6	0.25
Counterclockwise rotation						
time (sec)	13.2 ± 14.4	16.1 ± 29.7	0.62	12.1 ± 14.0	10.6 ± 10.9	0.87
number of steps	25.3 ± 13.7	23.5 ± 15.2	0.62	23.4 ± 12.2	22.3 ± 9.9	0.77
Data are presented as mean ± SD. Wilcoxon signed-rank sum test was used.						

The 30-second walking distance, which was the primary endpoint, showed no significant improvement in either SMA or M1 stimulation compared to sham stimulation (*p* = 0.49 and 0.23, respectively).

The results for secondary endpoints were as follows. In the 10-m natural walking and 360-degree rotation test, there were no significant differences in required time or number of steps for either SMA or M1 stimulation compared to sham stimulation.

In the TUG test, significant reduction was observed in required time (*p* = 0.03) or number of steps (*p* = 0.047) with SMA stimulation compared to sham stimulation. On the other hand, no significant difference was observed in required time (*p* = 0.49) or number of steps (*p* = 0.81) with M1 stimulation compared to sham stimulation.

### 3.3. Adverse event

No serious adverse events occurred. However, 10 patients (50%) complained of a tingling sensation on the scalp during stimulation. This event was reported by the participants shortly after the start of the real stimulation and assumed to be electrical stimulation through the scalp. This tingling sensation disappeared after stimulation ended. None of the participants experienced persistent symptoms such as scalp irritation. One patient showed bilateral lower extremity involuntary movement (dyskinesia) a few minutes after starting M1 stimulation, but the symptoms disappeared after stimulation ended.

## 4. Discussion

This pilot study investigated the effects of single-session anodal tDCS using a commercially available, wearable device on gait in PD patients with FOG. While neither SMA nor M1 stimulation significantly improved the primary outcome (30-second walking distance), SMA stimulation significantly reduced the time and number of steps in the TUG test compared to sham stimulation. These results suggest that SMA stimulation may enhance complex motor functions related to mobility, even if specific effects on FOG could not be confirmed.

SMA stimulation using this simple tDCS device was effective in improving gait only in the TUG test. The 30-second walking distance, 10-m natural walking, and 360-degree rotation test are evaluations of simple evaluation of walking distance, speed, and rotating ability. On the other hand, TUG test including straight-line walking, changes in direction, standing up and sitting down, evaluates these factors comprehensively. The TUG test measures the time required for a subject to get up from a chair, walk to a destination approximately 3 meters away, turn 180 degrees, and return to the same chair. The TUG test is highly reliable and highly correlated with daily life functions such as lower limb muscle strength, balance, walking ability, and falling. In general, the TUG test can predict the patient’s ability to go outside alone safely [43]. The present results suggest that SMA stimulation using this commercially available tDCS device may improve the practical walking ability of PD patients who exhibit FOG. Therefore, patients could use this wearable tDCS device in daily life and in gait rehabilitation [44,45].

The original purpose of this study was to verify the effect of tDCS on FOG. However, the findings only demonstrated the effect of SMA stimulation on the comprehensive walking ability evaluated by the TUG test, not on 30-second walking distance and 10-m natural walking. Therefore, no effect of SMA stimulation on FOG could be verified. In addition, some patients who complained of FOG did not show FOG during the gait evaluation, so only the general effect on walking function was verified.

SMA stimulation with tDCS improved performance in the TUG test as the SMA is involved in motor planning and coordination, so SMA activation may have improved motor control in PD patients. Lu et al reported that SMA stimulation with tDCS did not improve FOG in PD patients [28]. However, their protocol and testing were limited to the examination of the anticipatory postural adjustments and first step components of gait initiation, which are pure FOG examinations. In addition, the stimulation intensity was only 1 mA for 10 minutes, which may have been too weak. In our study, SMA stimulation was effective only in the TUG test. However, SMA stimulation may have improved other behaviors such as obstacle avoidance and direction changes rather than FOG.

Dagan et al demonstrated simultaneous tDCS stimulation of motor (M1) and cognitive (DLPFC) regions improved FOG and TUG test [25]. In fact, cognitive decline is known to be an independent risk factor for FOG [7], and stimulation of cognitive areas may also improve FOG. However, in our pilot study, all participants maintained their cognitive function evaluated by MMSE, FAB, and MoCA, so it seems that the influence of cognitive function on FOG was small.

The tDCS device used was not originally intended for medical use, but was developed to improve the performance of athletes. The use of tDCS by athletes has recently been identified as possible brain doping [46]. tDCS to the primary motor cortex enhances voluntary contraction and improves motor function, and has also been clinically applied for post-stroke rehabilitation or treatment of depression [47,48]. Since most tDCS devices are generally commercially available, patients can freely purchase and use such devices for rehabilitation in daily life. Therefore, any effect on gait in PD patients with FOG would show important potential as a treatment. The DCS device used in this study easily allowed stimulation of the bilateral M1 of the lower limb regions and the bilateral SMA.

Fortunately, no serious adverse events were observed with the use of this tDCS device. Half of the patients complained about the expected phenomenon of tingling sensation in the scalp [49]. On the other hand, one patient showed dyskinesia during M1 stimulation. Dyskinesia is usually caused by overdose of levodopa in PD patients. Therefore, M1 stimulation by tDCS may have similar effects to levodopa in PD patients. In fact, it is reported that M1 stimulation improved bradykinesia in PD patients [50]. We believe that these adverse effects are unlikely to affect the feasibility of daily use of this device.

These results encourage further investigation into the practical use of wearable neuromodulation devices in PD. Future research could explore personalized stimulation protocols, repeated-session effects, and combination with physical therapy to enhance therapeutic outcomes.

### 4.1. Strengths and limitations

A major strength of this study is the use of a sham-controlled, within-subject design, which minimizes interindividual variability and enhances internal validity. The use of a widely available, user-friendly tDCS device enhances the translational potential of our findings.

However, several limitations must be acknowledged. First, various factors may influence the actual effectiveness of tDCS on gait in PD patients. In fact, the effects of tDCS on gait ability varied widely from participant to participant in this study. In general, tDCS is not a pinpoint stimulation. The electrode size of the tDCS device is large, so accurate demarcation of the stimulation area of SMA and M1 is not possible. Thus, it is possible that stimulation of the SMA may partially extend to the M1 and surrounding areas, and vice versa. In addition, the effects of tDCS are modified by differences in the anatomy such as head size, skull thickness, subcutaneous fat, and cerebrospinal fluid cavity [51]. Therefore, the same intensity of tDCS stimulation can cause different responses in individuals [52]. Certain stimulations may be effective for some patients and not for others.

Besides, PD is a disease that shows a relatively strong placebo effect [53], and gait was improved to some extent with just sham stimulation in some patients. This phenomenon may have masked the actual effects of tDCS in some participants.

This study evaluated the walking ability in a spacious rehabilitation room with consideration for the safety of the participants. However, FOG is likely to occur in narrow spaces in daily life of PD patients [6]. Therefore, participants who complain of FOG in daily life may show little to no gait disturbance in controlled environments. In fact, some participants did not show obvious gait disturbance at the time of evaluation. Therefore, in future similar studies, it would be preferable to design the study in a narrow space, where FOG is easier to induce.

Motor fluctuations induced by levodopa medication are often seen in advanced PD patients. In principal, gait evaluation was performed in the medication-off period. However, sham stimulation was performed first, followed by real stimulation in this study protocol. In most patients, motor symptoms usually worsen over time unless next medications are taken. Therefore, it is possible that the baseline walking ability was decreased in the later evaluation with real stimulation compared to the earlier evaluation with sham stimulation. Nevertheless, we believe it is noteworthy that SMA stimulation showed significant improvement in the TUG test.

As for sample size, we recruited only 20 patients. The rationale for the sample size was described in the 2.1. Participants section. However, although no relevant data were available in the literature, it would have been possible to calculate the sample size based on some assumptions.

## 5. Conclusions

This pilot study demonstrated that SMA stimulation using a commercially available wearable tDCS device may improve the practical walking ability of PD patients who suffer FOG. It is expected that PD patients use this wearable tDCS device in their daily lives and gait rehabilitation. However, the effectiveness of this simple, non-invasive wearable device in treating freezing of gait may vary depending on the target patient. Future robust and longitudinal studies with larger cohorts is needed to validate the findings of this small pilot study.

## Supporting information

S1 File Data set. (XLSX)

S2 File Original study protocol in Japanese. (PDF)

S3 File English translation of the study protocol. (PDF)

S4 File TREND Statement Checklist. (DOCX)
