# Supplementary material for: Effects of transcranial direct current stimulation using a commercially available device on gait in Parkinson’s disease with freezing of gait
Source: PLoS One. 2025 Aug 21;20(8):e0330286. doi: 10.1371/journal.pone.0330286 (PMC12370039; doi:10.1371/journal.pone.0330286)
Supplement: S2 File — (PDF) [file pone.0330286.s002.pdf]

すくみ足に対する経頭蓋直流電気刺激の効果

研究計画書

研究責任医師
順天堂大学 運動障害疾患病態研究・治療講座、脳神経外科　　梅村淳

２０２０年９月８日　作成

略語および用語説明

略語および用語	説明
tDCS	transcranial direct current stimulation：経頭蓋直流電気刺激
SMA	supplementary motor area：補足運動野
M1	primary motor cortex：一次運動野

研究概要
（該当無いところは未記入で可）

研究の目的		すくみ足に対する経頭蓋直流電気刺激の効果を検証する	
試験のフェーズ		N/A	
Phase		N/A	
実施期間		jRCT公表日～2023年6月30日	
実施予定研究対象者数		20	
試験の種類		介入研究	
Study Type		Interventional	
試験デザイン		単一群／非盲検／プラセボ対照／単群比較／治療	
Study Design		Single arm study／open (masking not used)／placebo control／single assignment／treatment purpose	
プラセボの有無		☑ あり	☐ なし
盲検の有無		☐ あり	☑ なし
無作為化の有無		☐ あり	☑ なし
保険外併用療養の有無		☐ あり	☑ なし
臨床研究を実施する国(日本以外)		なし	
Countries of Recruitment		None	
研究対象者の適格基準<br>Key Inclusion & Exclusion Criteria	主たる選択基準	1. 20歳以上の男性および女性患者（年齢・性別を含む）<br>2. 順天堂大学医学部附属順天堂医院脳神経外科または脳神経内科に検査または治療目的で入院中で、パーキンソン病またはパーキンソン症候群と診断されている患者<br>3. すくみ足の症状がある患者<br>4. 本研究への参加にあたり十分な説明を受けた後、十分な理解の上、患者本人の自由意思による文書同意が得られた患者	
	Inclusion Criteria	1. Male and female patient aged 20 or older.<br>2. Patients diagnosed with Parkinson disease or Parkinson syndrome who are admitted to Department of Neurosurgery or Neurology, Juntendo Hospital, Juntendo University School of Medicine, for examination or treatment purpose.<br>3. Patients who suffer from freezing of gait.<br>4. Participants who have been given sufficient explanations to participate in this study and have given their full understanding and consent	
	主たる除外基準	1. 脳深部刺激装置、ペースメーカーや除細動器など植込み型の医療機器や電子機器を使用している患者<br>2. 関連のない臨床的に重大なうつ病など精神医学上の問題があると試験担当医師が判断する患者<br>3. 頭蓋骨の刺激部分にけがや欠損のある患者<br>4. てんかんやてんかん発作の既往がある患者<br>5. 頭蓋内に金属コイルを留置している患者<br>6. その他、研究責任者が被験者として不適当と判断した患者	
	Exclusion Criteria	1. Patients who use implantable medical or electronic devices such as deep brain stimulation, pacemakers and defibrillators<br>2. Patients who are judged by the investigator to have a psychiatric problem, such as unrelated clinically significant depression<br>3. Patients who have injuries or defects in the stimulated part of the skull<br>4. Patients who have a history of epilepsy or seizures<br>5. Patients who have a metal coil in the skull<br>6. Patients who the investigator judged to be inappropriate as subjects	
	年齢下限	20歳以上	

	Age Minimum	20 years old and over
	年齢上限	未設定
	Age Maximum	No setting
	性別	男性・女性
	Gender	Both
中止基準		＜研究対象者ごとの中止基準＞<br>1.　研究対象者（又は代諾者）より同意の撤回があった場合<br>2.　副作用など好ましくない事象が発現した場合<br>3.　その他の理由により、研究責任医師および研究分担医師が研究の中止が適当と判断した場合<br><br>＜臨床研究全体の中止基準＞<br>1.　認定臨床研究審査委員会が研究を継続すべきでないと判断した場合<br>2.　研究の安全性に疑義が生じた場合<br>3.　研究の倫理的妥当性や科学的妥当性を損なう事実や情報が得られた場合<br>4.　研究の実施の適正性や結果の信頼を損なう情報や事実が得られた場合
対象疾患名		パーキンソン病
Health Condition(s) or Problem(s) Studied		Parkinson disease
対象疾患キーワード		パーキンソン病
Keyword		Parkinson disease
介入の有無		☑　あり　　☐　なし
介入の内容		補足運動野ならびに一次運動野に対する経頭蓋直流電気刺激
Intervention(s)		Transcranial direct current stimulation to supplementary motor cortex and primary motor cortex
介入キーワード		経頭蓋直流電気刺激
Keyword		Transcranial direct current stimulation
主たる評価項目		＜PART1＞＜PART1＞<br>SMA 刺激と sham 刺激の 30 秒間歩行距離の比較<br>＜PART2＞<br>M1 刺激と sham 刺激の 30 秒間歩行距離の比較
Primary Outcome(s)		＜PART1＞<br>Comparison of walking distance for 30 seconds between SMA and sham stimulation<br>＜PART2＞<br>Comparison of walking distance for 30 seconds between M1 and sham stimulation
副次的な評価項目		10m自然歩行での歩行時間とステップ数<br>Timed Up and Go test<br>360° 回転試験
Secondary Outcome(s)		Walking time and number of steps in 10m walk<br>Timed Up and Go test<br>360 degree turn test

目次

1. 臨床研究の実施体制 1
2. 臨床研究の背景 2
3. 臨床研究の目的 3
4. 対象疾患 3
  4.1. 対象疾患 3
  4.2. 対象疾患の判断基準 3
5. 臨床研究の方法 3
  5.1. 臨床研究デザイン 3
  5.2. 臨床研究実施期間 3
  5.3. 臨床研究のアウトライン 3
6. 臨床研究の対象者の選択基準及び除外基準 4
  6.1. 選択基準 4
  6.2. 除外基準 4
7. 研究対象者の登録方法・割付方法 5
  7.1. 登録方法 5
  7.2. 割付方法 5
  7.3. 盲検化 5
8. 研究の中止基準 5
  8.1. 研究対象者ごとの中止基準 5
  8.2. 臨床研究全体の中止基準 5
9. 臨床研究の対象者に対する治療/介入 6
  9.1. 臨床研究に用いる医薬品等の概要 6
10. 投薬・手術・検査等の介入を行う手順 6
  10.1. 投薬部位・手術部位・検査部位等 6
  10.2. 投薬・手術・検査等の介入を行う時期・期間 7
  10.3. 用法・用量、回数、所要時間等 7
  10.4. 増量・減量の目安等 7
11. 併用薬及び併用療法 7
  11.1. 併用禁止薬及び禁止療法 7
  11.2. 併用可能薬・可能療法 7
12. 観察・検査項目及び実施時期，データ収集の方法 7
  12.1. 観察・検査スケジュール 7
  12.2. 観察・検査項目 8
  12.3. 観察・検査方法 8
13. 評価項目 9
  13.1. 有効性評価項目 9
    13.1.1. 主要評価項目（プライマリーエンドポイント） 9
    13.1.2. 副次的評価項目（セカンダリーエンドポイント） 9
  13.2. 安全性評価項目 9
14. 疾病等発生時の取り扱い 9
  14.1. 疾病等 9
    14.1.1. 疾病等の定義 9
  14.2. 予測される疾病等 9
  14.3. 予測できない疾病等 9
  14.4. 重篤度の判断 9
  14.5. 重篤な疾病等発生時の研究対象者への対応 10

14.6　疾病等の報告 10
14.7.　救済処置 10
14.7.1.　救済薬の交付、治療方法 10
14.7.2.　急性増悪等緊急時の処置 10
15.　統計学的事項 11
15.1.　目標症例数および設定根拠 11
15.2.　解析対象集団 11
15.3.　集計・解析方法 11
15.4.　欠落、不採用及び異常データの取扱いの手順 11
15.5.　当初の統計的な解析計画を変更する場合の手順 11
15.6.　中間解析と研究の早期中止 11
15.7. 11
15.5.　その他、探索的解析 11
16.　原資料等の閲覧 12
17.　品質管理及び品質保証 12
17.1.　モニタリング及び監査 12
17.1.1.　モニタリング 12
17.1.2.　監査 12
17.2.　データマネジメント 13
18.　倫理的な配慮 13
18.1.　遵守すべき諸規則 13
18.2.　研究対象者の個人情報及びプライバシーの保護 13
19.　臨床研究の対象者に対する説明及び同意を得る方法 14
19.1.　研究対象者に生じる負担並びに予測されるリスク及び利益の要約 14
19.2.　予測される利益 14
19.3.　予測される危険と不利益及びそれらを最小化する対策 14
19.4.　同意を得る手順 14
19.5.　同意説明文書の内容 14
20.　記録（データを含む。）の取扱い及び保存 15
20.1.　他機関への試料・情報の提供の有無 15
20.1.1　他機関への試料・情報の提供の有無 15
20.1.2　試料・情報の保管及び廃棄の方法 15
20.2.　研究に係る試料及び情報等の保管 15
20.3.　研究対象者から取得された試料・情報の二次利用について 15
21.　研究対象者の健康、子孫に受け継がれ得る遺伝的特徴等に関する重要な知見が得られた場合の研究対象者に係る研究結果（偶発的所見を含む。）の開示について 15
22.　臨床研究の実施に係る金銭の支払及び補償 16
22.1.　保険への加入の有無とその内容 16
22.2.　健康被害に対する補償・賠償 16
22.3.　予測される医療費（研究対象者の負担） 16
22.4.　研究対象者に対する金銭の支払、医療費の補助 16
23.　臨床研究に関する情報の公表 16
23.1.　研究に関する登録 16
23.2.　研究に関する情報の更新 16
23.3.　研究成果の帰属と結果の公表 16
24.　臨床研究の適正な実施のために必要な事項 16
24.1.　本臨床研究に対する医薬品等製造販売業者等による研究資金の提供等 16
24.2.　研究資金の拠出元 17

24.3.　利益相反 17
25.　症例報告書（CRF）の取り扱い 17
26.　研究実施計画書の改訂 17
27.　研究対象者等及びその関係者からの相談等への対応 17
28.　研究実施後における研究対象者への医療の提供に関する対応 17
29.　参考資料、文献リスト 17

1.　臨床研究の実施体制

■ 研究責任医師
梅村淳
順天堂大学運動障害疾患病態研究・治療講座、脳神経外科　特任教授
住所：〒113-8431　東京都文京区本郷 3-1-3
電話番号：03-3813-3111（内線 71008） E-mail：aumemura@juntendo.ac.jp

■ 研究分担医師
「研究分担医師リスト」参照

■ 研究事務局責任者
梅村淳
順天堂大学運動障害疾患病態研究・治療講座、脳神経外科　特任教授
住所：〒113-8431　東京都文京区本郷 3-1-3
電話番号：03-3813-3111（内線 71008） E-mail：aumemura@juntendo.ac.jp

■ 統計解析責任者
柳澤尚武
順天堂大学革新的医療技術開発研究センター　准教授
住所：〒113-8421　東京都文京区本郷 2-1-1
電話番号：03-3830-3704（内線 2109） E-mail：n-yanagisawa@juntendo.ac.jp

■ データマネジメント責任者
岩室宏一
順天堂大学運動障害疾患病態研究・治療講座、脳神経外科　准教授
住所：〒113-8431　東京都文京区本郷 3-1-3
電話番号：03-3813-3111（内線 70674） E-mail：h-iwamuro@juntendo.ac.jp

■ モニタリング責任者
菊地綾子
順天堂大学医学部附属順天堂医院　臨床研究・治験センター　臨床研究支援室
住所：〒113-8431　東京都文京区本郷 3-1-3
電話番号：03-3813-3111（内線 3832）

■ 監査責任者
藤林和俊
順天堂大学医学部附属順天堂医院　臨床研究・治験センター
臨床研究コンプライアンス・ガバナンス推進室　室長
住所：〒113-8431　東京都文京区本郷 3-1-3
電話番号：03-3813-3111（内線 3832）

■ 割り付け責任者（必要な場合）
割り付けなし

■ 医薬品管理者（医療機器管理者）
梅村淳
順天堂大学運動障害疾患病態研究・治療講座、脳神経外科　特任教授

住所:〒113-8431 東京都文京区本郷 3-1-3
電話番号:03-3813-3111(内線 71008) E-mail:aumemura@juntendo.ac.jp

■ 個人情報管理責任者
梅村淳
順天堂大学運動障害疾患病態研究・治療講座、脳神経外科 特任教授
住所:〒113-8431 東京都文京区本郷 3-1-3
電話番号:03-3813-3111(内線 71008) E-mail:aumemura@juntendo.ac.jp

■ 研究・開発計画支援担当者
該当無し

■ 調整管理実務担当者
該当無し

■ 研究代表医師・研究責任医師以外の研究を総括する者
該当無し

■ 臨床研究に関連する臨床検査施設、医学的及び技術的部門・機関
該当無し

■ 開発業務受託機関
該当無し

2. 臨床研究の背景

パーキンソン病(PD)は、振戦、固縮、無動、姿勢反射障害などの運動症状を主徴とする進行性の神経難病である。現時点で根治的な治療法は確立されていないものの、その病態は大脳基底核の機能異常とされており、対症療法としてドパミン作動性薬剤の補充療法や大脳基底核の機能異常を修飾するためのニューロモデュレーション(視床下核や淡蒼球に対する脳深部刺激療法(DBS))が行われている。その結果、運動症状の多くはコントロール可能となったが、未だに治療抵抗性の運動症状もあり今後の課題となっている。特に「すくみ足」は歩行開始時や方向転換時に足底があたかも床面にへばりついたようになって歩けなくなる状態で、PD および PS の運動症状の中でも最も厄介な症状の一つである。転倒の主因となり患者の日常生活動作を著しく低下させる要因となる。特に薬剤オン時のすくみ足は薬物療法や DBS が全く無効で、ドパミン以外のメカニズムが関与している可能性がある。その治療は困難を極め、新たな治療法の開発が望まれている。すくみ足の病態は未だ明らかになっていないが、歩幅と歩調の制御不全による歩行リズムの障害が根底にあり、その原因として近年 functional MRI による解析では、基底核のみならず大脳皮質(補足運動野や前頭葉)の神経活動の低下が指摘されている[1)]。

経頭蓋直流電気刺激(tDCS)は頭皮上に設置した電極から微弱な(0.5～2mA)直流電流が頭蓋骨を通過し、電極直下の皮質興奮性を修飾する非侵襲的刺激法である[2,3)]。tDCS はターゲットになる皮質領域に置く電極の極性により、脳機能を興奮性または抑制性に変化させることが可能である。すなわち陽極下では細胞膜が脱分極して皮質興奮性が増加し、陰極下では過分極が生じて興奮性が低下する。効果は刺激終了後も一定期間(数分から 1 時間程度)持続することからシナプスの可塑性をも変化させる可能性が指摘されている。さらに tDCS は刺激装置、電極とも比較的安価であり、小型軽量で運搬も容易であることから実際に患者を歩行させながら刺激することも可能である。一次運動野への tDCS は随意収縮を増強させ運動機能改善効果が報告されていることから近年本邦においても脳卒中後のリハビリテーションでの臨床応用が進められている。今回の研究では、tDCS による大脳皮質のニューロモデュレーション(補足運動野(SMA)刺激および一次運動野(M1)刺

激）がすくみ足を改善させうる可能性について検討する。本研究ではアスリートの運動機能向上を目的として一般に市販されている tDCS 装置（Halo Sport）を使用する[4,5]。この装置は比較的安価であり、小型軽量で実際に患者を歩行させながら刺激することも可能であることから、この装置を用いたすくみ足の治療やリハビリテーションへの応用を目指す。

3. 臨床研究の目的

経頭蓋直流電気刺激（tDCS）による補足運動野（SMA）または一次運動野（M1）に対するニューロモデュレーションがパーキンソン病のすくみ足を改善させうる可能性について検討する。本研究で一定の効果が得られた場合には、例えばこの方法をリハビリに組み合わせて行うといったような新規のすくみ足治療法の開発に繋げたい。

4. 対象疾患

4.1. 対象疾患

パーキンソン病

4.2. 対象疾患の判断基準

MDS診断基準に基づいて判断する。すなわち、パーキンソニズムとして運動緩慢がみられることが必須で、加えて静止時振戦か筋強剛のどちらか１つまたは両方がみられるものを対象とする。

5. 臨床研究の方法

5.1. 臨床研究デザイン

単一群／非盲検／プラセボ対照／単群比較／治療

5.2. 臨床研究実施期間

研究実施期間 :jRCT公表日～2023年6月30日
症例登録期間 :jRCT公表日～2022年12月31日

5.3. 臨床研究のアウトライン

本試験で行うtDCS刺激では実際に刺激を行うと患者は頭皮に違和感を感じるので刺激の有無を盲検化することは困難である。一方、刺激を行わない状態(sham刺激)でもtDCS装置を装着したことによる影響を排除するためにsham刺激、実刺激での評価を行う。一般にパーキンソン病患者は薬物療法により症状の日内変動が大きいので検査を行うタイミングによって患者の状態が異なる可能性がある。したがって刺激試験を行う際には、sham刺激、実刺激をセットで評価しないと適切な評価ができないと考える。

本研究においては、PART 1でsham刺激に対するSMA刺激の有効性及び安全性評価およびPART 2でsham刺激に対するM1刺激の有効性及び安全性評価の2つの試験を実施する。PART 1では最初に何も介入なしの状態で各種歩行評価の練習を行う。次いでtDCS装置を安静状態で20分頭部に装着(sham刺激:実際には刺激しない)した後、装置を取り外して歩行評価を行う。その後再度tDCS装置を装着して実際に2mAで20分間のSMA刺激を行なった後、装置を取り外して歩行評価を行う。ここまでをPART 1とする。この時点で個々の患者において患者状態や歩行評価の実施可能性を考慮してPART 2への移行の可否判定を行う。移行可能と判断された場合には、PART 1施行後6時間以上の間隔を空けてPART 2の試験を行う。PART 2でもPART 1と同様に最初に何も介入なしの状態で各種歩行評価の練習を行い、次いでtDCS装置を装着して20分間のsham刺激を行った後の歩行評価を行う。その後再度tDCS装置を装着して実際に2mAで20分間のM1刺激を行なって歩行評価を行いPART 2を終了する。

また、先行研究によりtDCSによる皮質興奮性の増加は刺激終了後も最大150分程度持続することが知られていることから、PART 1でのtDCS刺激の影響を完全に排除するために6時間以上の十分なウォッシュアウト期間をおいてからPART 2の試験を行う。

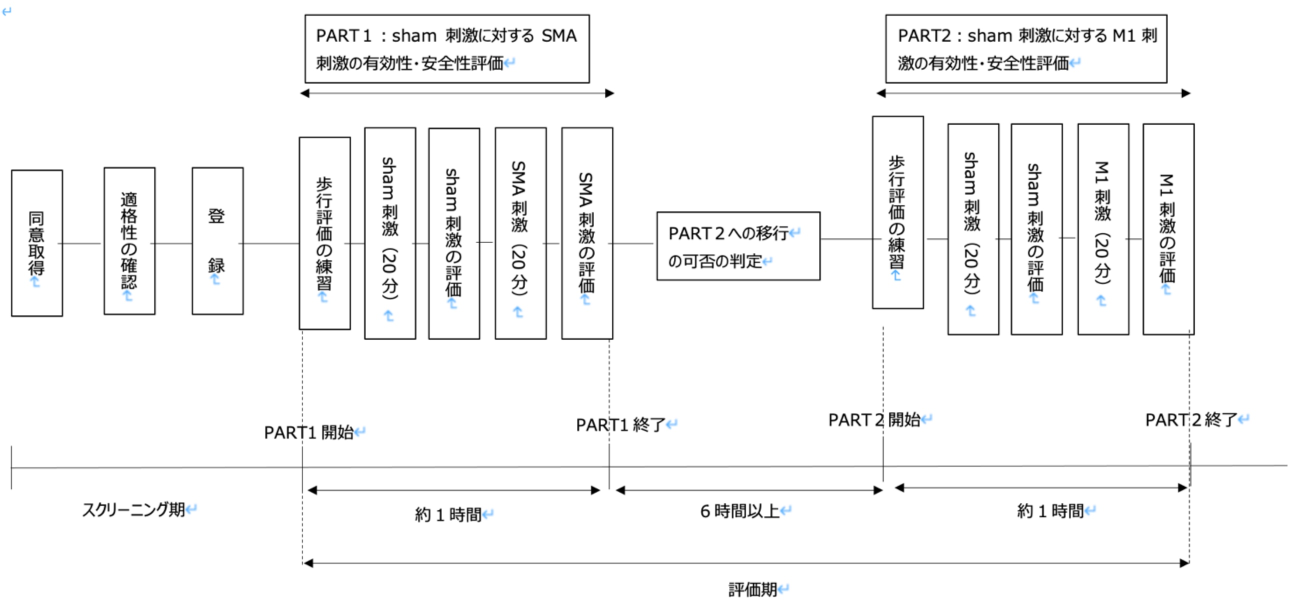

6. 臨床研究の対象者の選択基準及び除外基準

下記の選択基準を全て満たし、かつ除外基準のいずれにも該当しない研究対象者を対象とする。

6.1. 選択基準

1） 20 歳以上の男性および女性患者（年齢・性別を含む）
2） 順天堂大学医学部附属順天堂医院脳神経外科または脳神経内科に検査または治療目的で入院中で、パーキンソン病と診断されている患者
3） すくみ足の症状がある患者
4） 本研究への参加にあたり十分な説明を受けた後、十分な理解の上、患者本人の自由意思による文書同意が得られた患者

［設定根拠］

1） 未成年の患者では十分な理解が得られない可能性があり、対象疾患においてきわめて特異的であるため
2） パーキンソン病を対象とした臨床研究であるため
3） パーキンソン病のすくみ足に対する臨床研究であるため
4） 臨床研究において十分な理解が得られない患者や自由意志による同意が得られない患者は対象としない

6.2. 除外基準

1） 脳深部刺激装置、ペースメーカーや除細動器など植込み型の医療機器や電子機器を使用している患者
2） 関連のない臨床的に重大なうつ病など精神医学上の問題があると試験担当医師が判断する患者
3） 頭蓋骨の刺激部分にけがや欠損のある患者
4） てんかんやてんかん発作の既往がある患者
5） 頭蓋内に金属コイルを留置している患者
6） その他、研究責任者が被験者として不適当と判断した患者

［設定根拠］

1） 経頭蓋直流電気刺激が脳深部刺激装置、ペースメーカーや除細動器など植込み型の医療機器や電子機器に影響する可能性があるため
2） 経頭蓋直流電気刺激が臨床的に重大なうつ病など精神医学上の問題に対して影響する可能性があるため
3） 頭蓋骨の刺激部分にけがや欠損があると予定以上に強い刺激が加わる可能性があるため
4） 経頭蓋直流電気刺激がてんかん発作を誘発する可能性を否定できないため
5） 経頭蓋直流電気刺激と金属コイルとの相互作用の可能性を否定できないため
6） 研究責任医師が被験者として不適当と判断した患者は対象としない

6.3. PART2 への移行判定基準

PART1実施後の患者の全身状態、有害事象の有無を確認した上で、PART2の実施は問題ないと研究責任医師または研究分担医師が判断した場合

7. 研究対象者の登録方法・割付方法

7.1. 登録方法

1） 本研究への登録に際し、研究責任医師または研究分担医師は患者に対し本研究に関する十分な説明を口頭及び文書で行った上で、研究参加の同意を文書で取得する。同意書は2部取得し、そのうち1部は患者へ返却し、残りの1部は医療機関の担当診療科にて保管する。
2） 同意取得後、適格性確認を行い、対象患者が選択基準を全て満たし、除外基準の何れにも該当しないことが確認された症例を被験者として登録する。
3） 症例登録は大学内で管理する EDC である REDCap を用いて、研究責任医師または研究分担医師が行う。
4） 症例登録管理責任者は岩室宏一（順天堂大学運動障害疾患病態研究・治療講座、脳神経外科 准教授）とする。

7.2. 割付方法

本試験では割付は行わない。

7.3. 盲検化

被験者に対して刺激の有無は伝え盲検化は行わない。

8. 研究の中止基準

8.1. 研究対象者ごとの中止基準

以下のような場合には研究を中止する。研究を中止した場合は、その理由を明らかにして、登録用紙に記入する。

1） 研究対象者（又は代諾者）より同意の撤回があった場合
2） 副作用など好ましくない事象が発現した場合
3） その他の理由により、研究責任医師および研究分担医師が研究の中止が適当と判断した場合

8.2. 臨床研究全体の中止基準

下記に該当した場合は研究全体を中止する。研究責任医師は、研究を中止した場合には、研究対象者に中止したことを速やかに通知し、適切な医療の提供やその他の必要な措置を講ずる。研究責任医師は、研究を中止したときには、中止及びその理由、結果概要を文書により遅滞なく病院長に報告する。

1） 認定臨床研究審査委員会が研究を継続すべきでないと判断した場合
2） 研究の安全性に疑義が生じた場合
3） 研究の倫理的妥当性や科学的妥当性を損なう事実や情報が得られた場合

4）　研究の実施の適正性や結果の信頼を損なう情報や事実が得られた場合

9.　臨床研究の対象者に対する治療/介入

9.1.　臨床研究に用いる医薬品等の概要

経頭蓋直流電気刺激装置（tDCS 装置）

販　売　名：Halo Sport 2

製　造　元：Halo Neuroscience 社

概　　　要：一般に市販されているヘッドホン型の tDCS 装置である。3 組の刺激パッドが装着されており、これを頭頂部（M1）に当てると正中のパッドは左右両側の上下肢並びに体幹の、両側のパッドは左右の手指領域の anode 刺激を行うように設計されている。もともとアスリートのトレーニングや楽器のスキル向上のために開発されたデバイスで、最大出力は 2.0mA で、操作はスマホアプリで行い、1 回 20 分以上の刺激ができない仕様となっている。さらに小型軽量で実際に患者を歩行させながら刺激することも可能である。

今回の研究では通常の頭頂部への装着で正中のパッドからの刺激で両側の上下肢並びに体幹の M1 刺激が可能で、その状態から 2～3cm 前の冠状縫合のあたりにずらすことで両側 SMA 刺激が可能となる。Sham 刺激においてはそれぞれの部位に合わせて装置を装着するのみで実際に刺激は行わない。

重大な副作用：本装置は実際に一般に市販されている tDCS 装置であり、広く一般に使用されており安全性は刺激装置の設計によって担保されている。本装置による刺激は、我が国の臨床神経生理学会の委員会からの 3mA の強度で 30 分までの刺激は安全であるという提言の範囲内で行われるので重大な副作用をきたす可能性は少ないと思われる。しかしながらその副作用として、頭皮に多少のうずきを感じることは予想される。

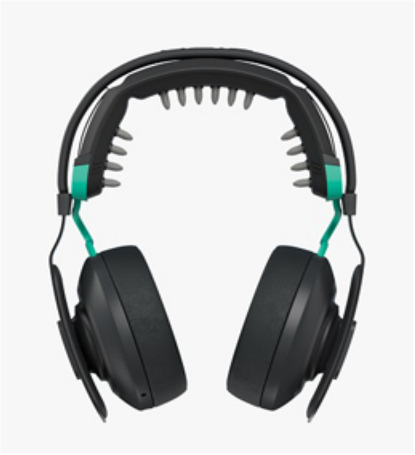

Halo Sport 2

10.　投薬・手術・検査等の介入を行う手順

10.1.投薬部位・手術部位・検査部位等

刺激部位は補足運動野（SMA）および一次運動野（M1）上の頭皮で、それぞれ sham 刺激と実刺激を行う。

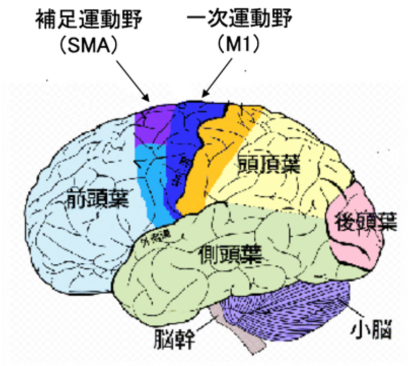

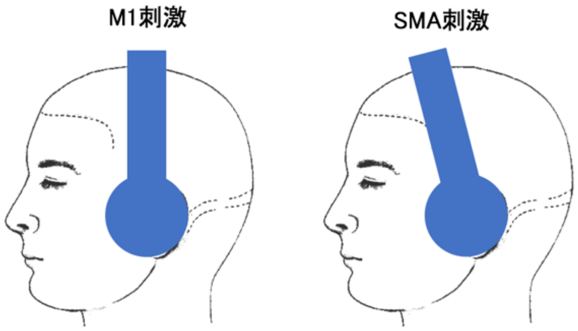

10.2.投薬・手術・検査等の介入を行う時期・期間

本登録後、1～2 日間で歩行評価を 2 セット（PART1 では 2 回の歩行評価、PART2 では 2 回の歩行評価：計 6 回）を行う。1 セット目の歩行評価終了後は、6 時間以上の wash out 期間を設ける。

10.3.用法・用量、回数、所要時間等

最初にtDCS装置を装着せず何も行わない状態で歩行評価の練習を行う。次いでtDCS装置を安静状態で20分装着（sham刺激）した後、装置を取り外して歩行評価を行う。その後再度tDCS装置を装着して実際に2mAで20分間の皮質刺激を行なった後、装置を取り外して歩行評価を行う。この3回の評価を1セッションとし、6時間以上の間隔を空けて刺激部位を変えて同様の評価を行う。刺激部位は最初（PART1）に補足運動野（SMA）とし、2回目（PART2）は一次運動野（M1）とする。1回の試験所要時間は約1時間程度である。

症状変動の大きなパーキンソン病患者の場合にはできる限り薬剤オフ状態（薬が切れて症状が重い状態すなわちパーキンソン症状が目立つ状態）での評価を行う。ただし実際に服用している薬剤を中止することは無い。

10.4.増量・減量の目安等

刺激強度や刺激時間は一定とするが、刺激が不快な場合には刺激強度を下げることで対応する。

11. 併用薬及び併用療法

11.1.併用禁止薬及び禁止療法

併用禁止薬は特に定めない。

11.2.併用可能薬・可能療法

疾患に対する薬物療法はすべて可能とし、実際に服用している薬剤を中止することは無い。

12. 観察・検査項目及び実施時期，データ収集の方法

12.1.観察・検査スケジュール

実施期間	スクリーニング期	評価期		
		PART 1	PART2 への移行の判定	PART 2
実施時点		スクリーニング終了後任意*の時期	PART1 終了後 6 時間以内	PART 1 終了後 6 時間以上経過後
被験者基本情報の確認	○			

<table>
<tr><td colspan="2">適格性の確認</td><td>○</td><td></td><td>○</td><td></td></tr>
<tr><td colspan="2">同意取得</td><td>○</td><td></td><td></td><td></td></tr>
<tr><td colspan="2">MDS-UPDRS</td><td>○</td><td></td><td></td><td></td></tr>
<tr><td colspan="2">認知機能評価
（MMSE, FAB, MoCA-J）</td><td>○</td><td></td><td></td><td></td></tr>
<tr><td colspan="2">QOL 評価（PDQ-39）</td><td>○</td><td></td><td></td><td></td></tr>
<tr><td colspan="2">すくみ足の評価（FOG-Q）</td><td>○</td><td></td><td></td><td></td></tr>
<tr><td colspan="2">Sham 刺激／SMA 刺激</td><td></td><td>○</td><td></td><td></td></tr>
<tr><td rowspan="5">Sham 刺激後／SMA 刺激後</td><td>30 秒間での
歩行距離</td><td></td><td>○</td><td></td><td></td></tr>
<tr><td>10m 自然歩行
時間とステップ数</td><td></td><td>○</td><td></td><td></td></tr>
<tr><td>Timed Up and
Go test</td><td></td><td>○</td><td></td><td></td></tr>
<tr><td>360° 回転試験</td><td></td><td>○</td><td></td><td></td></tr>
<tr><td>歩行状態の
ビデオ撮影</td><td></td><td>○</td><td></td><td></td></tr>
<tr><td colspan="2">Sham 刺激/M1 刺激</td><td></td><td></td><td></td><td>○</td></tr>
<tr><td rowspan="5">Sham 刺激後／M1 刺激後</td><td>30 秒間での
歩行距離</td><td></td><td></td><td></td><td>○</td></tr>
<tr><td>10m 自然歩行
時間とステップ数</td><td></td><td></td><td></td><td>○</td></tr>
<tr><td>Timed Up and
Go test</td><td></td><td></td><td></td><td>○</td></tr>
<tr><td>360° 回転試験</td><td></td><td></td><td></td><td>○</td></tr>
<tr><td>歩行状態の
ビデオ撮影</td><td></td><td></td><td></td><td>○</td></tr>
<tr><td colspan="2">有害事象・不具合</td><td></td><td>○</td><td>○</td><td>○</td></tr>
</table>

＊スクリーニング終了後 3 ヶ月以内（一旦退院した後に再入院する場合も対象とする）

12.2.観察・検査項目

① 研究対象者基本情報：年齢、性別、疾患の治療歴
② MDS-UPDRS
③ 認知機能評価：MMSE, FAB, MoCA-J
④ QOL 評価：PDQ-39
⑤ すくみ足の評価：freezing of gait questionnaire（FOG-Q）
⑥ 歩行能力の評価：30 秒間での歩行距離、10m 自然歩行時間とステップ数、Timed Up and Go test
⑦ 歩行状態のビデオ撮影

12.3.観察・検査方法

① 研究対象者基本情報：年齢、性別、PD の治療歴→カルテからの抜粋
② MDS-UPDRS→脳神経内科医による診察
③ 認知機能評価：MMSE, FAB, MoCA-J→脳神経内科医による評価
④ QOL 評価：PDQ-39→質問票による
⑤ すくみ足の評価：freezing of gait questionnaire（FOG-Q）→質問票による
⑥ 歩行能力の評価：30 秒間での歩行距離、10m 自然歩行時間とステップ数、Timed Up and Go test、360° 回転試験→リハビリ室で実施

⑦ 歩行状態のビデオ撮影→リハビリ室で被験者により実施

13. 評価項目

13.1.有効性評価項目

13.1.1. 主要評価項目（プライマリーエンドポイント）

＜PART1＞

SMA 刺激と sham 刺激の 30 秒間歩行距離の比較

＜PART2＞

M1 刺激と sham 刺激の 30 秒間歩行距離の比較

【設定根拠】

主要評価項目の設定根拠として、すくみ足を認める患者では歩行速度が低下するが、重症例では歩行時に全く足が出ずにほとんど歩行距離が出せないこともある。このような場合でも歩行距離を主要評価項目とすればデータとして残すことができる。一方、副次評価項目に設定した 10m 歩行時間はこのような重症例ではデータを得ることができずにその患者を除外せざるを得ない可能性がある。したがって歩行距離を主要評価項目に設定した。

13.1.2. 副次的評価項目（セカンダリーエンドポイント）

10m 自然歩行時間とステップ数

Timed Up and Go test

360°回転試験

13.1.3. 探索的評価項目

歩行状態のビデオ撮影による客観的評価

13.2. 安全性評価項目

有害事象、不具合の発生状況

14. 疾病等発生時の取り扱い

14.1.疾病等

14.1.1. 疾病等の定義

疾病等とは、臨床研究の実施に起因するものと疑われる疾病、障害若しくは死亡または感染症に加え、臨床検査値の異常や諸症状を含むものをいう。

14.2.予測される疾病等

研究対象者が刺激を行う頭皮にうずきや痛痒感、頭痛、灼熱感などの不快感を感じる可能性がある。また、歩行評価中に転倒による打撲、骨折等の可能性がある。

14.3.予測できない疾病等

「14.2.予測される疾病等」の項で指定された以外の疾病等。

14.4.重篤度の判断

医療機器の不具合の発生であって、当該不具合によって次に掲げる疾病等が発生するおそれのあるものについて知ったときは、これを知った日から三十日以内にその旨を実施医療機関の管理者に報告した上で、当該実施計画に記載された認定臨床研究審査委員会に報告しなければならない。

（1） 死亡

（2） 死亡につながるおそれのある疾病等

(3) 治療のために医療機関への入院又は入院期間の延長が必要とされる疾病等

(4) 障害

(5) 障害につながるおそれのある疾病等

(6) (3)～(5)まで並びに死亡及び死亡につながるおそれのある疾病等に準じて重篤である疾病等

(7) 後世代における先天性の疾病又は異常

14.5.重篤な疾病等発生時の研究対象者への対応

研究責任医師は、疾病等を認めた場合には、直ちに適切な処置を行う。

なお、本研究の実施に伴い、研究対象者に健康被害が発生した場合の補償責任に備え、当研究は臨床研究保険に加入する。万が一、本研究への参加に起因して重い健康被害（死亡、後遺障害 1 級・2 級）が生じた場合には当該保険から補償の給付を受けることができる。

14.6 疾病等の報告

研究責任医師は、本研究の実施において疾病等の発生を知った場合には、速やかに、その旨を実施医療機関の管理者に報告した上で、認定臨床研究審査委員会へ報告する。

認定臨床研究審査委員会が疾病等の報告に対し、意見を述べた時は、研究責任医師は、当該意見を尊重して必要な措置をとる。

なお、実施医療機関の管理者及び認定臨床研究審査委員会への報告は、以下の期間内に行う。

①以下の疾病等の発生のうち、未承認又は適応外の医薬品等を用いる特定臨床研究の実施によるものと疑われるものであって予測できないもの　7 日

(a) 死亡

(b) 死亡につながるおそれのある疾病等

②以下の疾病等の発生のうち、未承認又は適応外の医薬品等を用いる特定臨床研究の実施によるものと疑われるもの（①に掲げるものを除く）　15 日

(a) 死亡

(b) 死亡につながるおそれのある疾病等

③以下の疾病等の発生のうち、未承認又は適応外の医薬品等を用いる特定臨床研究の実施によるものと疑われるものであって予測できないもの　15 日

(a) 治療のために医療機関への入院又は入院期間の延長が必要とされる疾病等

(b) 障害

(c) 障害につながるおそれのある疾病等

(d) (a)から(c)まで並びに死亡及び死亡につながるおそれのある疾病等に準じて重篤である疾病等

(e) 後世代における先天性の疾病又は異常

④上記①～③以外については、定期報告（実施計画を提出した日から起算して、1 年ごとに、当該期間満了後 2 か月以内）の際に行う。

14.7.救済処置

14.7.1. 救済薬の交付、治療方法

研究対象者が痛痒感、頭痛、灼熱感などの不快感を訴えた場合には直ちに刺激を中止する。

14.7.2. 急性増悪等緊急時の処置

直ちに刺激を中止するとともに直ちに適切な処置を行う。

15. 統計学的事項

15.1.目標症例数および設定根拠

目標症例数 20 例

本試験は探索的な試験のため、統計学的検出力に基づいて目標症例数を算出するだけの十分な情報がない。従って、当院における年間の当該疾患入院患者数（約 500 名）から選択基準を満たし、かつ研究参加同意を得られる患者を推定（月 1 例程度）し、研究期間（2 年 6 か月）内での実施可能数である 20 例とする。

15.2.解析対象集団

本試験の有効性解析対象集団は、原則としてすべての症例を対象とした、Intention-to-treat (ITT) 解析対象集団とするが、以下の被験者は解析対象集団から除く。

1）組み入れ基準を満たしていない者

2）一度も、装置をつけて測定しなかった者。

一度でも tDCS 刺激を行った被験者を含む対象集団を、安全性解析対象集団として解析を実施する。

15.3.集計・解析方法

研究対象者背景は、連続変数は平均値及び標準偏差、カテゴリカル変数に関しては、頻度と割合を算出して集計する。連続変数が明らかに正規分布に従わない場合は、変数を対数変換などで適切に変換し、平均値および標準偏差で集計するか、または、中央値、四分位範囲を記述統計量として使用する。

主要評価項目である、tDCS 刺激後と sham 刺激後の 30 秒間での歩行距離の差について、1標本 t 検定またはウィルコクソン符号付順位検定で評価する。

副次評価項目である、tDCS 刺激後と sham 刺激後の 10m 自然歩行時間およびステップ数、Timed Up and Go test、360° 回転試験の差について、1標本 t 検定またはウィルコクソン符号付順位検定で評価する。

ならびに

歩行状態のビデオ撮影については、改善が得られた場合において専門家（神経内科医、リハビリテーション医）の視点から歩行状態の変化を客観的に解析するために行う。

安全性については、有害事象ごとに頻度と割合（全被験者に対する。）を算出し、集計する。

15.4.欠落、不採用及び異常データの取扱いの手順

何らかの理由により本研究計画書に記載された検査ならびにデータ収集が行えなかった症例については欠落、不採用とし、原則欠落値の補完は行わない。また、外れ値などの異常値について、原則すべてのデータを解析に用いるが、除外場合は、除外理由を記録する。欠落値および異常データの取り扱いについては、統計解析計画書に記載する。

15.5.当初の統計的な解析計画を変更する場合の手順

当初の統計的な解析計画からの変更がある場合は、研究計画書又は統計解析計画書を改訂し、臨床研究の総括報告書においても説明する。

15.6.中間解析と研究の早期中止

本試験において中間解析は行わない。

15.7.その他、探索的解析

試験の結果によっては、サブグループ解析などの探索的な解析を実施する場合がある。解析目的、項目、手法、結果については、最終報告書に記載する。

16. 原資料等の閲覧

研究責任医師及び実施医療機関は、臨床研究に関連するモニタリング、監査並びに認定臨床研究審査委員会及び規制当局による調査の際に、原資料等すべての臨床研究関連記録を直接閲覧に供する。

17. 品質管理及び品質保証

17.1.モニタリング及び監査

17.1.1. モニタリング

<u>実施体制</u>

本研究のモニタリングは、研究責任医師がモニター指定書により指定した順天堂大学医学部附属順天堂医院 臨床研究・治験センターに所属する者が実施する。

連絡先：〒113-8431 東京都文京区本郷 3-1-3
TEL（直通） 03-3814-5672 （内線） 3832

<u>モニタリングの方法</u>

本研究のモニタリングは、中央モニタリングにより実施する。モニターは、モニタリング実施後、1 ヶ月以内にモニタリング報告書を作成し、研究責任医師に提出する。

〇モニタリング対象施設

順天堂大学医学部附属順天堂医院 脳神経外科
順天堂大学医学部附属順天堂医院 脳神経内科

<u>モニタリングの実施時期</u>

本研究のモニタリングは、研究終了時に 1 回実施する

<u>モニタリングの確認項目</u>

モニターは、以下の事項を確認する。

① 症例登録状況：登録数、累積
② 適格性：不適格例/不適格の可能性のある患者
③ 治療前背景因子（研究対象者基本情報）
④ 試験治療中/治療終了の別、中止例、その理由
⑤ 不適合の有無、その内容
⑥ 疾病等、機器の不具合の有無、その内容、対応状況
⑦ その他、試験の進捗や安全性に関する問題点

17.1.2. 監査

<u>実施体制</u>

本研究の監査は、順天堂大学医学部附属順天堂医院 臨床研究・治験センター 臨床研究コンプライアンス・ガバナンス推進室 室長の指名により、研究責任医師が監査員指定書をもって指定した者が実施する。

連絡先：〒113-8431 東京都文京区本郷 3-1-3
TEL(直通) 03-3814-5672 (内線)3832

<u>監査の実施方法</u>

(1) 本研究の監査は、施設訪問監査により実施する。

〇監査対象施設

・順天堂大学医学部附属順天堂医院 脳神経外科
・順天堂大学医学部附属順天堂医院 脳神経内科

(2) 直接閲覧（SDV）は、実施医療機関の手順等の定めに従って実施する。

監査の実施時期

本研究の監査は、本研究の終了時及びその他必要が生じた場合に実施する。

監査員は、監査終了後から１ヶ月以内に監査報告書を研究責任医師に提出する。

〇研究終了時に実施する監査

本研究のモニタリングが終了した後、監査を実施する。

〇必要が生じた場合に実施する監査

本研究において必要が生じた場合に実施する監査は、以下とする。

（ア）疾病等報告を厚生労働大臣に提出した場合

（イ）法、施行規則又は研究計画書に対する重大な不適合※が発覚した場合

（ウ）その他、研究責任医師が必要と判断した場合

※重大な不適合とは、対象者の人権、安全性、研究の進捗、結果の信頼性に影響を及ぼすもの（例：選択・除外基準、中止基準等の不遵守等）。対象者の緊急の危険を回避するためその他医療上やむを得ない理由により研究計画書に従わなかったものは含まない。

監査の確認項目

本研究の監査は、以下に定める項目を確認する。

・臨床研究審査委員会の手続き関連書類
・研究対象者より取得した同意書、同意撤回書
・研究対象者識別コードリスト
・個人情報の保管・管理状況
・症例の適格性の確認
・受診、検査の実施状況
・研究中止例数、中止理由
・疾病等、機器不具合の有無、内容、対応状況
・臨床研究法又は研究計画書に対する不適合（重大な不適合を含む）の有無、内容、対応状況
・モニタリングの実施結果、モニタリング報告書の保管状況
・その他、研究責任医師が必要とする事項

17.2.データマネジメント

本研究では、CRF 及びマネジメントツールとして REDCap を用いてデータマネジメントを行う。データ固定後に、統計解析責任者である柳澤が統計解析を行う。

18. 倫理的な配慮

18.1.遵守すべき諸規則

本研究に携わるすべての者は、人を対象とする全ての医学研究が準拠すべき「世界医師会ヘルシンキ宣言」、「人を対象とする医学系研究に関する倫理指針」及び「臨床研究法」の内容を熟読し理解した上で遵守し、研究を施行する。

18.2.研究対象者の個人情報及びプライバシーの保護

研究に関わる関係者は、研究対象者の個人情報保護について、適用される法令、条例を遵守する。 また関係者は、研究対象者の個人情報及びプライバシー保護に最大限の努力を払い、本研究を行う上で知り得た個人情報を正当な理由なく漏らしてはいけない。関係者がその職を退いた後も同様とする。

研究実施に係る個人情報を取扱う際は、各参加施設の(個人情報管理者等)によって、個人情報とは関係ない研究用IDを付して管理し、研究対象者の秘密保護に十分配慮する。作成した対応表は、脳神経外科研究室の鍵のかかるロッカーで研究責任者が厳重に管理する。

19. 臨床研究の対象者に対する説明及び同意を得る方法

19.1.研究対象者に生じる負担並びに予測されるリスク及び利益の要約

本研究に参加することで研究対象者に新たな経済的負担が生じることは無い。

研究対象者が本研究に参加することによって生じると予測される直接的な利益はなく、不利益についても同様である。

19.2.予測される利益

研究対象者が本試験に参加することで意味のある利益を受けるかどうかは不明であるが、今回の研究成果により、現在有効な治療法が無い難治性のすくみ足の改善効果やリハビリテーションへの応用が期待でき、将来の医療の進歩に貢献できる可能性がある。

本研究で試験を行う治療において特に新たなコストが発生することはない。また、研究対象者の研究期間中の薬剤費を含む診療費はすべて患者の保険及び患者自己負担により支払われるため、研究対象者が研究に参加することで得られる、特別な診療上、経済上の利益はない。

19.3.予測される危険と不利益及びそれらを最小化する対策

経頭蓋直流電気刺激は、我が国の臨床神経生理学会の委員会からの 3mA の強度で 30 分までの刺激は安全であるという提言に基づき、刺激はこの範囲内で行われる。実際に本研究で使用する刺激装置は実際に一般に市販されている装置であり、最大出力は 2.0mA で 1 日 20 分以上の刺激ができない仕様となっている。

しかしながらその有害事象として、頭皮に多少のうずきを感じることは予想される。刺激が不快な場合には刺激強度を下げることで対応する。また、被験者が痛痒感、頭痛、灼熱感などの不快感を訴えた場合には直ちに刺激を中止する。

19.4.同意を得る手順

研究責任医師、研究分担医師は、研究対象者に対して別に定める説明・同意文書に基づき、本臨床研究に参加する前に研究の内容について十分に説明する。

なお、説明・同意文書は研究責任医師が作成し、認定臨床研究審査委員会の承認を得た後に使用する。改訂する場合は再度認定臨床研究審査委員会に申請し、承認を得た後に使用する。

臨床研究に参加するかどうかについて十分考える時間を与えた後、研究責任医師及び研究分担医師は本人の自由意思による研究参加の同意を文書(別途定める同意文書)で得る。

19.5.同意説明文書の内容

同意文書に記載する項目は以下の通りとする。

1) 臨床研究の名称及び当該臨床研究の実施について研究機関の長の許可を受けている旨及び厚生労働大臣に実施計画を提出している旨
2) 研究機関の名称及び研究責任者の氏名及び職名
(他の研究機関と共同して研究を実施する場合には、研究代表医師の氏名及び職名並びに共同研究機関の名称及び共同研究機関の研究責任者の氏名び職名を含む。)
3) 臨床研究の目的及び意義
4) 臨床研究の方法(研究対象者から取得された試料・情報の利用目的を含む。)及び期間
5) 研究対象者として選定された理由
6) 研究対象者に生じる負担並びに予測されるリスク及び利益
7) 臨床研究が実施又は継続されることに同意した場合であっても随時これを撤回できる旨(研究対象者等からの撤回の内容に従った措置を講じることが困難となる場合があるときは、その旨及びその理由)
8) 臨床研究が実施又は継続されることに同意しないこと又は同意を撤回することによって研究対象者等が不利益な取扱いを受けない旨

9) 臨床研究に関する情報公開の方法
10) 研究対象者又はその代諾者の求めに応じて、他の研究対象者等の個人情報等の保護及び当該研究の独創性の確保に支障がない範囲内で研究計画書及び臨床研究の方法に関する資料を入手又は閲覧できる旨並びにその入手又は閲覧の方法
11) 個人情報等の取扱い（匿名化する場合にはその方法、匿名加工情報又は非識別加工情報を作成する場合にはその旨を含む。）
12) 試料・情報の保管及び廃棄の方法
13) 臨床研究の資金源等、研究機関の研究に係る利益相反及び個人の収益等、研究者等の研究に係る利益相反に関する状況
14) 研究対象者等及びその関係者からの苦情及び問合せへ等への対応
15) 研究対象者等に経済的負担又は謝礼がある場合には、その旨及びその内容
16) 通常の診療を超える医療行為を伴う臨床研究の場合には、他の治療方法等に関する事項
17) 通常の診療を超える医療行為を伴う研究の場合には、研究対象者への研究実施後における医療の提供に関する対応
18) 臨床研究の実施に伴い、研究対象者の健康、子孫に受け継がれ得る遺伝的特徴等に関する重要な知見が得られる可能性がある場合には、研究対象者に係る研究結果（偶発的所見を含む。）の取扱い
19) 侵襲を伴う臨床研究の場合には、当該研究によって生じた健康被害に対する補償の有無及びその内容
20) 研究対象者から取得された試料・情報について、研究対象者等から同意を受ける時点では特定されない将来の研究のために用いられる可能性又は他の研究機関に提供する可能性がある場合には、その旨と同意を受ける時点において想定される内容
21) 侵襲（軽微な侵襲を除く。）を伴う臨床研究であって介入を行うものの場合には、研究対象者の秘密が保全されることを前提として、モニタリングに従事する者及び監査に従事する者並びに認定臨床研究審査委員会が、必要な範囲内において当該研究対象者に関する試料・情報を閲覧する旨

20. 記録（データを含む。）の取扱い及び保存

20.1.他機関への試料・情報の提供の有無

20.1.1 他機関への試料・情報の提供の有無

☐ あり
☑ なし

20.1.2 試料・情報の保管及び廃棄の方法

本研究は他機関への試料・情報を提供しないため該当なし。

20.2.研究に係る試料及び情報等の保管

研究責任医師は、研究等の実施に係わる文書（申請書類の控え、病院長からの通知文書、各は種申請書・報告書の控、研究対象者識別コードリスト、同意書、症例報告書等の控、その他データの信頼性を保証するのに必要な書類又は記録など）を保存し、研究終了後 5 年間に保存する。

廃棄する場合には、個人が特定できないよう、匿名化したまま廃棄する。

20.3.研究対象者から取得された試料・情報の二次利用について

本研究で得られたデータについては、認定臨床研究審査委員会の審査を経て承認された場合に限り、個人識別情報とリンクしない形で二次利用することがあり得る。

21. 研究対象者の健康、子孫に受け継がれ得る遺伝的特徴等に関する重要な知見が得られた場

合の研究対象者に係る研究結果（偶発的所見を含む。）の開示について

本研究は、研究対象者の健康、子孫に受け継がれ得る遺伝的特徴等に関する研究ではないため、該当なし。

22. 臨床研究の実施に係る金銭の支払及び補償

22.1.保険への加入の有無とその内容

☑ 加入する

☐ 加入しない

＜加入する場合、その内容＞

臨床研究賠償責任保険

22.2.健康被害に対する補償・賠償

本研究の実施に伴い、研究対象者に健康被害が発生した場合の補償責任に備え、当臨床研究は臨床研究保険に加入する。万が一、本研究への参加に起因して重い健康被害（死亡、後遺障害 1 級・2 級）が生じた場合には研究者の加入する保険から補償の給付を受けることができる。

また、各研究対象者の研究終了後、当該研究の結果により得られた最善の医療（予備、診断及び治療）を受けることができるよう努力する。

22.3.予測される医療費（研究対象者の負担）

本研究に参加することによる研究対象者の費用負担は発生しない。一般保険診療に該当する負担のみ生じる。

22.4.研究対象者に対する金銭の支払、医療費の補助

本研究に参加することによる研究対象者に対する金銭の支払、医療費の補助は無い。

23. 臨床研究に関する情報の公表

23.1.研究に関する登録

研究に関する情報は、厚生労働省が設置している公開データベース：jRCT（Japan Registry of Clinical Trials、URL: https://jrct.niph.go.jp/）に登録する

23.2.研究に関する情報の更新

jRCT に登録した情報は、適宜、一更新等を行う。

23.3.研究成果の帰属と結果の公表

本研究で得られた結果は、パーキンソン病・運動障害学会で発表し、運動障害領域の専門学術誌で論文として公表する予定である。いずれの場合においても公表する結果は統計的な処理を行ったものだけとし、研究対象者の個人情報は一切公表しない。

研究代表医師は、主要評価項目データの収集期間が終了した日から 1 年以内に主要評価項目報告書を、全てのデータの収集期間が終了した日から 1 年以内に総括報告書・総括報告書の概要をそれぞれ作成し、認定臨床研究審査委員会の意見を聴いた日から起算して 1 月以内に jRCT に公開する。

24. 臨床研究の適正な実施のために必要な事項

24.1.本臨床研究に対する医薬品等製造販売業者等による研究資金の提供等

☐ あり

☑ なし

＜ありの場合、その内容＞

24.2.研究資金の拠出元

本研究では、日本学術振興会から支給される科学研究費研究助成金を使用する。

研究種目名:基盤研究(C)(一般)

課題番号: 19K09465

研究課題名: パーキンソン病のすくみ足に対する経頭蓋直流電気刺激によるニューロモデュレーション

24.3.利益相反

本研究では、【日本学術振興会科学研究費から支給される研究助成金】を使用する。利益相反の管理については、研究責任(代表)医師が臨床研究法における臨床研究の利益相反管理ガイダンスに従い、利益相反管理基準及び利益相反管理計画を認定臨床研究審査委員会に提出し承認を得ている。

25. 症例報告書(CRF)の取り扱い

研究責任医師は、研究等の実施に係わる重要な文書(症例報告書(CRF):研究の対象者ごとに医薬品等を用いた日時及び場所等に関する記録、研究計画書、実施計画、本研究の対象者に対する説明及びその同意に係る文書、総括報告書、認定臨床研究審査委員会から受け取った審査意見業務に係る文書、モニタリング及び監査に関する文書、本研究の実施に係る契約書、本研究に用いる医薬品等の概要を記載した文書、その他本研究を実施するために必要な文書)の保存については、研究の中止または終了後 5 年が経過した日までの間、各実施医療機関の研究責任医師が定める場所にて保存し、その後は個人情報に注意して廃棄する。CRF の取り扱い詳細に関しては、データマネジメントマニュアルに規定する。最終的な研究結果は順天堂大学に帰属する。

26. 研究実施計画書の改訂

臨床研究を安全に実施する上で必要な情報を収集し、検討する。また、新たな安全性情報等が得られた場合、必要に応じて研究計画書および同意説明文書を変更する。研究計画書や同意説明文書に改訂の必要が生じた場合は、認定臨床研究審査委員会の定める手順に則って改訂する。

改訂の記録・理由などについては、表紙に示されている「更新・承認履歴一覧」に記載していく。

27. 研究対象者等及びその関係者からの相談等への対応

この臨床研究に関する相談窓口を以下のとおり設ける。

【相談窓口】

研究責任医師:順天堂大学医学部附属順天堂医院脳神経外科 特任教授 梅村淳

〒113-8431 東京都文京区本郷 3-1-3 順天堂大学医学部附属順天堂医院脳神経外科

電話番号:03-3813-3111 (内線 71008) E-mail:aumemura@juntendo.ac.jp

28. 研究実施後における研究対象者への医療の提供に関する対応

研究終了後は、通常の保険診療での治療を継続する。

29. 参考資料、文献リスト

1) Zhou C, Zhong X, Yang Y, Yang W, Wang L, Zhang Y, Nie K, Xu J, Huang B: Alterations of regional homogeneity in freezing of gait in Parkinson's disease. J Neurol Sci 387: 54-59, 2018
2) 緒方勝也, 飛松省三:経頭蓋直流電気刺激(tDCS)の基礎と臨床応用. 計測と制御 54: 106-113, 2015
3) 桐本光, 大西秀明:経頭蓋直流電気刺激を利用した中枢神経興奮性の修飾とその臨床応用. 理学療法学 44: 166-177, 2017
4) Edwards DJ, Cortes M, Wortman-Jutt S, Putrino D, Bikson M, Thickbroom G, Pascual-Leone A:

Transcranial direct current stimulation and sports performance. Front Hum Neurosci 11: 243, 2017
5) Huang L, Deng Y, Zheng X, Liu Y: Transcranial direct current stimulation with halo sport enhances repeated sprint cycling and cognitive performance. Front Hum Neurosci 10: 118, 2019
