# Supplementary material for: Effects of transcranial direct current stimulation using a commercially available device on gait in Parkinson’s disease with freezing of gait
Source: PLoS One. 2025 Aug 21;20(8):e0330286. doi: 10.1371/journal.pone.0330286 (PMC12370039; doi:10.1371/journal.pone.0330286)
Supplement: S3 File — (PDF) [file pone.0330286.s003.pdf]

Effect of transcranial direct current stimulation on freezing of gait

Study Protocol

Principal investigator
Department of Neurosurgery, Department of Research and Therapeutics for Movement Disorders, Juntendo University
Atsushi Umemura

Updated: Sep. 8, 2020

Abbreviations and glossary

Abbreviations and terms	explanation
tDCS	Transcranial Direct Current Stimulation
SMA	Supplementary motor area
M1	Primary motor cortex

Research Summary

(If not applicable, you can leave it blank.)

Purpose of the study		To examine the effect of transcranial direct current electrical stimulation on freezing of gait	
Phase		N/A	
Implementation Period		jRCT publication date - June 30, 2023	
Number of Participants		20	
Study Type		Interventional	
Study Design		Single arm study／open (masking not used)／placebo control／ single assignment／treatment purpose	
Placebo or not		☑ And	□ None
Blinding or not		□ Yes	☑ not
Presence or absence of randomization		□ Yes	☑ no
Whether or not there is uninsured concomitant medical treatment		□ Yes	☑ not
Countries of Recruitment other than Japan		None	
Eligibility Criteria for Study Subjects<br>Key Inclusion & Exclusion Criteria			
	Inclusion Criteria	1. Male and female patient aged 20 or older.<br>2. Patients diagnosed with Parkinson disease or Parkinson syndrome who are admitted to Department of Neurosurgery or Neurology, Juntendo Hospital, Juntendo University School of Medicine, for examination or treatment purpose.<br>3. Patients who suffer from freezing of gait.<br>4. Participants who have been given sufficient explanations to participate in this study and have given their full understanding and consent	
	Exclusion Criteria	1. Patients who use implantable medical or electronic devices such as deep brain stimulation, pacemakers and defibrillators<br>2. Patients who are judged by the investigator to have a psychiatric problem, such as unrelated clinically significant depression<br>3. Patients who have injuries or defects in the stimulated part of the skull<br>4. Patients who have a history of epilepsy or seizures<br>5. Patients who have a metal coil in the skull<br>6. Patients who the investigator judged to be inappropriate as subjects	
	Age Minimum	20 years old and over	
	Age Maximum	No setting	
	Gender	Both	
Discontinuation Criteria		<Discontinuation criteria for each study subject><br>1. When the consent of the research subject (or the agent) is withdrawn<br>2. When undesirable events such as side effects occur<br>3. When the principal investigator and co-investigator deem it appropriate to discontinue the study for any other reason.<br><br><Discontinuation criteria for the entire clinical study><br>1. When the Accredited Clinical Research Review Board determines that the research should not be continued.<br>2. If there is any doubt about the safety of the study<br>3. When facts or information are obtained that impair the ethical validity or scientific validity of the research<br>4. When information or facts are obtained that undermine the appropriateness of the conduct of the research or the credibility of the results	
Health Condition(s) or Problem(s) Studied		Parkinson's disease	
Target Disease Keywords		Parkinson's disease	
Intervention		Yes	
Intervention(s)		Transcranial direct current stimulation to supplementary motor cortex and	

	primary motor cortex
Keyword	Transcranial direct current stimulation
Primary Outcome(s)	<PART1><br>Comparison of walking distance for 30 seconds between SMA and sham stimulation<br><PART2><br>Comparison of walking distance for 30 seconds between M1 and sham stimulation
Secondary Outcome(s)	Walking time and number of steps in 10m walk<br>Timed Up and Go test<br>360 degree turn test

table of contents

1. Clinical Research Implementation System 1
2. Background of clinical research 2
3. Objectives of clinical research 3
4. Target Diseases 3
 4.1. Target Diseases 3
 4.2. Criteria for Determining Target Diseases 3
5. Methods of clinical research 3
 5.1. Clinical study design 3
 5.2. Duration of clinical research 3
 5.3. Clinical Research Outline 3
6. Selection and exclusion criteria for clinical research subjects 4
 6.1. Criterias of choice 4
 6.2. Exclusion Criteria 5
7. How to register and allocate research subjects 5
 7.1. How to register 5
 7.2. Randomization method 5
 7.3. Blinding 5
8. Study Discontinuation Criteria 6
 8.1. Discontinuation criteria per study subject 6
 8.2. Discontinuation criteria for the entire clinical study 6
9. Treatment for Subjects of Clinical Research/intervention 6
 9.1. Overview of Pharmaceuticals Used in Clinical Research 6
10. Procedures for administering interventions such as medication, surgery, and tests 7
 10.1. Medication site, surgery site, examination site, etc. 7
 10.2. Timing and duration of interventions such as medication, surgery, and examinations 7
 10.3. Usage, dose, frequency, time required, etc. 7
 10.4. Guidelines for weight increase and weight loss, etc. 8
11. Concomitant medications and concomitant therapies 8
 11.1. Concomitant Prohibited Drugs and Prohibited Therapies 8
 11.2. Concomitant drugs and therapies 8
12. Observation and inspection items, implementation timing, and data collection method 8
 12.1. Observation and Inspection Schedule 8
 12.2. Observation and Inspection Items 9
 12.3. Observation and Inspection Methods 9
13. Evaluation items 9
 13.1. Efficacy endpoints 9
  13.1.1. Primary endpoint (primary endpoint) 9
  13.1.2. Secondary endpoint (secondary endpoint) 10
 13.2. Safety endpoints 10
14. Handling in the event of an outbreak of illness 10
 14.1. Diseases, etc. 10
  14.1.1. Definition of Disease, etc. 10
 14.2. Predicted diseases, etc. 10
 14.3. Unpredictable diseases, etc. 10
 14.4. Determination of severity 10
 14.5. Responding to research subjects in the event of an outbreak of a serious illness 10

14.6 Reporting of illnesses, etc. 11
14.7. Remedies 11
14.7.1. Delivery of salvage drugs, methods of treatment 11
14.7.2. Emergency treatment such as acute exacerbation 11
15. Statistical matters 11
15.1. Target number of cases and rationale for setting 11
15.2. Population to be analyzed 11
15.3. Aggregation and Analysis Methods 12
15.4. Procedures for handling missing, rejected, and abnormal data 12
15.5. Procedure for modifying the original statistical analysis plan 12
15.6. Interim analysis and early discontinuation of the study 12
15.7. 12
15.5. Other, Exploratory Analysis 12
16. Viewing of original documents, etc. 12
17. Quality Control & Quality Assurance 13
17.1. Monitoring & Auditing 13
17.1.1. monitoring 13
17.1.2. audit 13
17.2. Data Management 14
18. Ethical Considerations 14
18.1. Rules to be observed 14
18.2. Protection of personal information and privacy of research subjects 14
19. Explanation and method of obtaining consent from the subject of clinical research 15
19.1. Summary of the burden and anticipated risks and benefits to the study subjects 15
19.2. Projected Profits 15
19.3. Anticipated Dangers and Disadvantages and Measures to Minimize Them 15
19.4. Steps to obtain consent 15
19.5. Contents of the Consent Briefing Document 15
20. Records (including data) Handling and storage of 16
20.1. Whether or not samples and information are provided to other organizations 16
20.1.1 Whether or not samples and information are provided to other organizations 16
20.1.2 Methods of storage and disposal of samples and information 16
20.2. Storage of samples and information related to research 17
20.3. Secondary Use of Samples and Information Obtained from Research Subjects 17
21. Results of research on research subjects (including accidental findings) when important findings were obtained regarding the health of the research subjects, genetic characteristics that can be passed on to offspring, etc. Disclosure of 17
22. Payment of money and compensation for the conduct of clinical research 17
22.1. Whether or not you have insurance and what it is 17
22.2. Compensation and reparation for health damage 17
22.3. Projected medical expenses (burden on the study subject) 17
22.4. Payment of money to research subjects, subsidy of medical expenses 17
23. Publication of information on clinical research 17
23.1. Register for research 17
23.2. Updates on research information 18
23.3. Attribution of research results and publication of results 18
24. Matters necessary for the proper conduct of clinical research 18
24.1. Provision of research funds by pharmaceutical manufacturers, distributors, etc. for this clinical

research 18
24.2. Sources of research funding 18
24.3. Conflict of Interest 18
25. Handling of Case Report Forms (CRFs) 18
26. Revision of the Research Implementation Plan 19
27. Responding to consultations from research subjects and related parties 19
28. Measures related to the provision of medical care to research subjects after the conduct of the research 19
29. References, bibliography 19

1. Clinical Research Implementation System

- Principal investigator
  Atsushi Umemura
  Specially Appointed Professor, Department of Neurosurgery, Department of Research and Therapeutics for Movement Disorders, Juntendo University
  Address: 〒113-8431 3-1-3 Hongo, Bunkyo-ku, Tokyo
  Phone number: 03-3813-3111 (ext. 71008) E-mail: aumemura@juntendo.ac.jp

- Co-investigator
  See "List of Principal Investigators"

- Head of Research Secretariat
  Atsushi Umemura
  Specially Appointed Professor, Department of Neurosurgery, Department of Research and Therapeutics for Movement Disorders, Juntendo University
  Address: 〒113-8431 3-1-3 Hongo, Bunkyo-ku, Tokyo
  Phone number: 03-3813-3111 (ext. 71008) E-mail: aumemura@juntendo.ac.jp

- Head of Statistical Analysis
  Naotake Yanagisawa
  Associate Professor, Center for Innovative Medical Technology Development, Juntendo University
  Address: 〒113-842 12-1-1 Hongo, Bunkyo-ku, Tokyo
  Phone number: 03-3830-3704 (ext. 2109) E-mail: n-yanagisawa@juntendo.ac.jp

- Head of Data Management
  Koichi Iwamuro
  Associate Professor, Department of Neurosurgery, Department of Research and Therapeutics for Movement Disorders, Juntendo University
  Address: 〒113-8431 3-1-3 Hongo, Bunkyo-ku, Tokyo
  Phone number: 03-3813-3111 (ext. 70674) E-mail: h-iwamuro@juntendo.ac.jp

- Person in charge of monitoring
  Ayako Kikuchi
  Juntendo University Hospital, Clinical Research and Clinical Trial Center, Clinical Research Support Office
  Address: 〒113-8431 3-1-3 Hongo, Bunkyo-ku, Tokyo
  Phone number: 03-3813-3111 (ext. 3832)

- Audit Officer
  Kazutoshi Fujibayashi
  Juntendo University School of Medicine Juntendo Hospital Clinical Research and Clinical Trial Center
  General Manager, Clinical Research Compliance and Governance Promotion Office
  Address: 〒113-8431 3-1-3 Hongo, Bunkyo-ku, Tokyo
  Phone number: 03-3813-3111 (ext. 3832)

- Person responsible for allocation (if required)
  No Allocation

- <u>Drug Administrator (Medical Device Administrator)</u>
  Atsushi Umemura

Specially Appointed Professor, Department of Neurosurgery, Department of Research and Therapeutics for Movement Disorders, Juntendo University
Address: 〒113-8431 3-1-3 Hongo, Bunkyo-ku, Tokyo
Phone number: 03-3813-3111 (ext. 71008) E-mail: aumemura@juntendo.ac.jp

■ Person in charge of personal information management
Atsushi Umemura
Specially Appointed Professor, Department of Neurosurgery, Department of Research and Therapeutics for Movement Disorders, Juntendo University
Address: 〒113-8431 3-1-3 Hongo, Bunkyo-ku, Tokyo
Phone number: 03-3813-3111 (ext. 71008) E-mail: aumemura@juntendo.ac.jp

■ R&D Planning Support Officer
Not applicable

■ Coordination Management Practitioner
Not applicable

■ A person who supervises research other than the principal investigator and principal investigator
Not applicable

■ Clinical laboratories, medical and technical departments and institutions related to clinical research
Not applicable

■ Contract Development Organizations
Not applicable

2. Background of clinical research

Parkinson's disease (PD) is a progressive intractable neurological disease characterized mainly by motor symptoms such as tremor, rigidity, akinesia, and postural reflex disorders. Although no curative treatment has been established at this time, the pathology is considered to be abnormal function of the basal ganglia, and as symptomatic treatment, replacement therapy of dopaminergic drugs and neuromodulation to modify the functional abnormality of the basal ganglia. Deep brain stimulation (DBS) therapy for the subthalamic nucleus and globus pallidus has been done. As a result, many of the motor symptoms have become controllable, but there are still treatment-resistant motor symptoms, which are an issue for the future. In particular, "freezing of gait" is a condition in which the sole of the foot becomes stuck to the floor at the start of walking or when changing direction, and is one of the most troublesome symptoms of PD. It is a major cause of falls and a factor that significantly reduces the patient's activities of daily living. In particular, when the drug is on, drug therapy and DBS are completely ineffective, and mechanisms other than dopamine may be involved. Treatment is extremely difficult, and the development of new treatments is desired. Although the pathophysiology of slurping feet has not yet been clarified, it is rooted in impaired gait rhythm due to incontrol of stride length and gait, and in recent years functional MRI analysis has pointed out a decrease in neural activity not only in the basal ganglia but also in the cerebral cortex, supplementary motor cortex and frontal lobe[1)].

Transcranial direct current stimulation (tDCS) is a non-invasive stimulation method in which a weak (0.5 to 2 mA) direct current passes through the skull from an electrode placed on the scalp and modifies cortical excitability directly under the electrode[2,3)]. tDCS can alter brain function to excitatory or inhibitory depending on the polarity of the electrodes placed in the targeted cortical region. That is, under the anode, the cell membrane is depolarized and cortical excitability increases, and under the cathode, hyperpolarization occurs and excitability decreases. Since the effect persists for a certain period of time (from a few minutes to about 1 hour) after the stimulation ends, it has been pointed out that it may also

change the plasticity of synapses. In addition, tDCS is relatively inexpensive for both stimulators and electrodes, and since it is compact, lightweight, and easy to transport, it is possible to stimulate the patient while actually walking. Since tDCS to the primary motor cortex has been reported to enhance voluntary contractions and improve motor function, clinical application in post-stroke rehabilitation has been promoted in Japan in recent years. In this study, tDCS is used to neuromodulate the cerebral cortex (supplementary motor cortex (SMA) stimulation and primary motor cortex (M1).We will examine the possibility that stimulation can improve freezing of gait. In this study, we used a commercially available tDCS device (Halo Sport) to improve the motor function of athletes[4,5)]. Since this device is relatively inexpensive, compact and lightweight, and can be stimulated while actually walking, we aim to apply this device to the treatment and rehabilitation of freezing of gait.

3. Objectives of clinical research

We will examine the possibility of that neuromodulation to the supplementary motor cortex (SMA) or primary motor cortex (M1) by transcranial direct current stimulation (tDCS) improves freezing of gait in Parkinson's disease. If a certain effect is obtained from this research, we would like to lead to the development of a new treatment method for freezing of gait, for example, by combining this method with rehabilitation.

4. Target Diseases

4.1. Target Diseases

Parkinson's disease

4.2. Criteria for Determining Target Diseases

Judgment will be made based on MDS diagnostic criteria. In other words, it is essential to have bradykinesia as parkinsonism, and in addition, one or both of resting tremor or muscle stiffness are observed.

5. Methods of clinical research

5.1. Clinical study design

Single-arm/open-label/placebo-controlled/single-arm comparison/treatment

5.2. Duration of clinical research

Research period: jRCT publication date to June 30, 2023
Period of patient registration: jRCT publication date to December 31, 2022

5.3. Clinical Research Outline

In the tDCS stimulation used in this study, it is difficult to blind the presence or absence of irritation because the patient feels discomfort in the scalp when the stimulation is actually performed. On the other hand, in order to eliminate the effects of wearing the tDCS device even in a state where no stimulation is performed (sham stimulation), evaluation is performed with sham stimulation and actual stimulation. In general, patients with Parkinson's disease have large motor fluctuations in symptoms due to drug therapy, so the patient's condition may vary depending on the timing of the examination. Therefore, when conducting a stimulation test, it is considered that an appropriate evaluation cannot be performed unless the sham stimulation and the actual stimulus are evaluated as a set.

In this study, we evaluated the efficacy and safety of SMA stimulation for sham stimulation in PART 1 and the efficacy and safety evaluation of M1 stimulation for sham stimulation in PART 2. Two tests will be conducted. In PART 1, participants will first practice various gait assessment without any intervention. Next, the tDCS device is worn on the head for 20 minutes in a resting state (sham stimulation: no actual stimulation), and then the device is removed and gait evaluation is performed. Thereafter, the tDCS device is attached again, SMA stimulation is actually performed at 2 mA for 20 minutes, and then the

device is removed and gait evaluation is performed. This is PART 1. At this point, a decision is made on whether or not to move to PART 2 in consideration of the patient's condition and the feasibility of gait evaluation for each patient. If it is judged that it is possible to migrate, the test of PART 2 will be conducted at an interval of at least 6 hours after the implementation of PART 1. In PART 2, as in PART 1, various gait evaluations are first practiced without any intervention, and then gait evaluation is performed after 20 minutes of sham stimulation with a tDCS device. After that, the tDCS device is attached again, and M1 stimulation is actually performed at 2 mA for 20 minutes, gait evaluation is performed, and PART 2 is completed.

In addition, since previous studies have shown that the increase in cortical excitability by tDCS persists for up to 150 minutes after the end of stimulation, a sufficient washout period of at least 6 hours should be given before PART 1 is completely eliminated before PART 2 tests conduct.

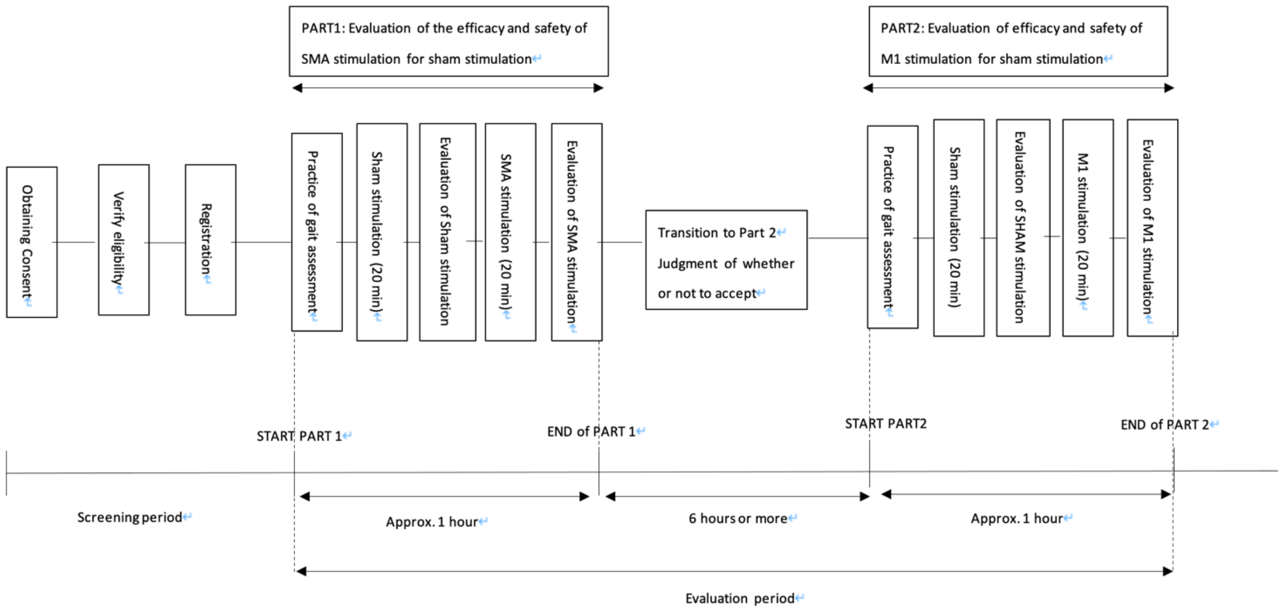

6. Selection and exclusion criteria for clinical research subjects

Study subjects who meet all of the following inclusion criteria and do not meet any of the exclusion criteria are included.

6.1. Criterias of choice

1) Male and female patients 20 years of age and older (including age and gender)
2) Patients who have been diagnosed with Parkinson's disease who are hospitalized at the Department of Neurosurgery or the Department of Neurology, Juntendo University Hospital for examination or treatment
3) Patients with symptoms of freezing of gait
4) Patients who have received a sufficient explanation for their participation in this study and have obtained written consent of their own free will after sufficient understanding

[Rationale]

1) It may not be fully understood in minor patients and is highly specific to the disease of interest.
2) Because it is a clinical study targeting Parkinson's disease
3) Because it is a clinical study for freezing of gait in Parkinson's disease
4) Patients who do not have a good understanding in clinical studies or who do not have free

consent are not included

6.2. Exclusion Criteria

1) Patients with implantable medical devices or electronic devices such as deep brain stimulators, pacemakers or defibrillators
2) Patients who are judged by the study physician to have unrelated clinically significant depression or other psychiatric problems
3) Patients with injuries or defects in the irritated part of the skull
4) Patients with a history of epilepsy or epileptic seizures
5) Patients with intracranial metal coils
6) Other patients who are judged by the principal investigator to be unsuitable as subjects

[Rationale]

1) Transcranial direct current electrical stimulation may affect implantable medical devices and electronic devices such as deep brain stimulators, pacemakers, and defibrillators.
2) Transcranial direct current electrical stimulation may affect psychiatric problems such as clinically significant depression
3) If there is an injury or defect in the irritated part of the skull, it may cause stronger stimulation than planned.
4) Because it cannot be ruled out that transcranial direct current electrical stimulation can induce epileptic seizures
5) Since the possibility of interaction between transcranial direct current electrical stimulation and metal coils cannot be ruled out
6) Patients deemed unsuitable as subjects by the principal investigator are not included

6.3. Criteria for Transition to PART 2

When the principal investigator or co-investigator determines that there is no problem with the implementation of PART 2 after confirming the patient's general condition and the presence or absence of adverse events after PART 1

7. How to register and allocate research subjects

7.1. How to register

1) Upon registration in this study, the principal investigator or co-investigator shall provide the patient with a sufficient explanation of this study orally and in writing, and obtain written consent to participate in the study. Two copies of the consent form are obtained, one of which is returned to the patient, and the other copy is kept at the department in charge of the medical institution.
2) After obtaining consent, eligibility will be confirmed, and cases that are confirmed to meet all of the inclusion criteria and do not fall under any of the exclusion criteria will be enrolled as subjects.
3) Patient registration will be conducted by the principal investigator or co-investigator using REDCap, an EDC managed by the university.
4) The person in charge of case registration management shall be Koichi Iwamuro (Associate Professor, Department of Neurosurgery, Department of Movement Disorder Disease Research and Treatment, Juntendo University).

7.2. Randomization method

No assignment was performed in this study.

7.3. Blinding

Subjects will be told whether or not they are stimulated and will not be blinded.

8. Study Discontinuation Criteria

8.1. Discontinuation criteria per study subject

Discontinue the study in the following cases: If you discontinue the study, clarify the reason and fill out the registration form.

1) When the consent of the research subject (or the agent) is withdrawn
2) When undesirable events such as side effects occur
3) When the principal investigator and co-investigator deem it appropriate to discontinue the study for any other reason.

8.2. Discontinuation criteria for the entire clinical study

In the following cases, the entire study will be discontinued. In the event that the study is discontinued, the investigator shall promptly notify the study subject of the discontinuation and take other necessary measures to provide appropriate medical care. When the principal investigator discontinues the research, the principal investigator shall report the discontinuation, the reason, and a summary of the results in writing to the hospital director without delay.

1) When the Accredited Clinical Research Review Board determines that the research should not be continued.
2) If there is any doubt about the safety of the study
3) When facts or information are obtained that impair the ethical validity or scientific validity of the research
4) When information or facts are obtained that undermine the appropriateness of the conduct of the research or the credibility of the results

9. Treatment/Intervention for Clinical Study Subjects

9.1. Overview of Pharmaceuticals Used in Clinical Research

Transcranial Direct Current Electrical Stimulator (tDCS Instrument)

Brand name : Halo Sport 2

manufacturer : Halo Neuroscience

Overview : It is a commercially available headphone-type tDCS device. Three pairs of stimulation pads are attached, which are attached to the crown of the head (M1). The midline pad is on the upper and lower limbs on both the left and right sides, as well as the trunk. The pads on both sides are designed to perform anode stimulation in the left and right hand finger areas. Originally for athlete training and musical instrumentsIt is a device developed to improve skills, has a maximum output of 2.0 mA, and is operated with a smartphone application.1timeIt is designed so that stimulation cannot be done for more than 20 minutes.In addition, it is small and lightweight, and it is possible to stimulate the patient while actually walking.

In this study, with normal attachment to the crown of the head, stimulation from the midline pad M1 stimulation of the upper and lower limbs on both sides and the trunk is possible, and from that state both sides SMA stimulation is possible by shifting in front of 3 cm around the coronal suture. In sham stimulation, All you have to do is attach the device to each part. It doesn't actually stimulate.

Significant side effects: This device is actually a commercially available tDCS device, which is widely used by the general public, and its safety is ensured by the design of the stimulator. Stimulation with this device is performed within the scope of the recommendation from the committee of the Japanese Society of Clinical Neurophysiology that stimulation at an intensity of 3 mA for up to 30 minutes is safe, so it is unlikely to cause serious side effects. However, as a side effect, it is expected that you will feel some tingling in the scalp.

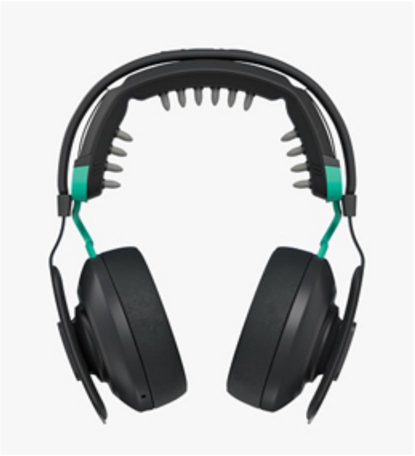

Halo Sport 2

10. Procedures for administering interventions such as medication, surgery, and tests

10.1. Medication site, surgery site, examination site, etc.

The stimulation sites are the scalp on the supplementary motor cortex (SMA) and the primary motor cortex (M1), respectively, for sham stimulation and actual stimulation.

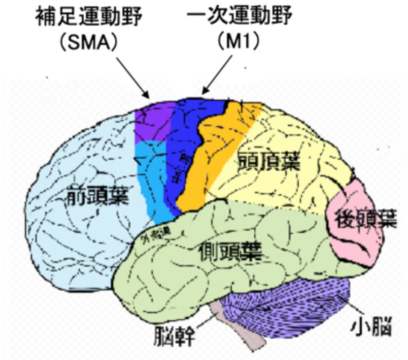

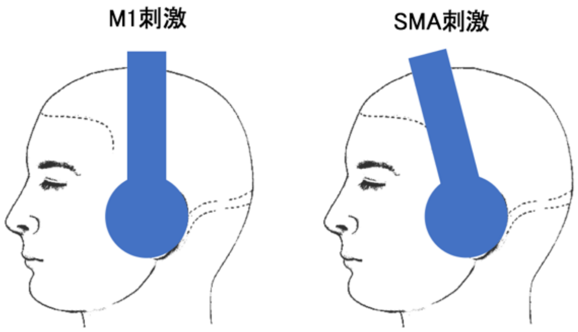

10.2. Timing and duration of interventions such as medication, surgery, and examinations

After this registration, 2 sets of walking evaluations will be performed in 1 to 2 days (2 walking evaluations in PART 1 and 2 walking evaluations in PART 2, 4 times in total). After the first set of walking evaluations, there will be a wash out period of at least 6 hours.

10.3. Usage, dose, frequency, time required, etc.

First, practice of in front of 3 cm gait assessment without wearing the tDCS device and without doing anything. Next, the tDCS device is worn in a resting state for 20 minutes (sham stimulation), and then the device is removed and gait evaluation is performed. Thereafter, the tDCS device is attached again and cortical stimulation is actually performed at 2 mA for 20 minutes, and then the device is removed and gait evaluation is performed. These two evaluations will be one session, and the same evaluation will be performed at different stimulation sites at intervals of 6 hours or more. The stimulation site is the supplementary motor cortex (SMA) in the first (PART1) and the second (PART2) is the primary motor cortex (M1). The duration of one test is about 1 hour.

In the case of patients with Parkinson's disease with large motor fluctuations, evaluation should be performed in a state where the drug is off (that is, when the symptoms are severe after the drug wears

off, that is, Parkinson's symptoms are noticeable). However, there is no discontinuation of the medication that is actually being taken.

10.4. Guidelines for stimulation increase and decrease, etc.

The intensity and duration of stimulation should be constant, but if the stimulus is unpleasant, the stimulation intensity will be reduced.

11. Concomitant medications and concomitant therapies

11.1. Concomitant Prohibited Drugs and Prohibited Therapies

There is no specific prohibition of concomitant drugs.

11.2. Concomitant drugs and therapies

All medications for the disease are possible, and there is no discontinuation of the medication that is actually being taken.

12. Observation and inspection items, implementation timing, and data collection method

12.1. Observation and Inspection Schedule

<table>
<tr><th colspan="2" rowspan="2">Implementation Period</th><th rowspan="2">Screening period</th><th colspan="3">Evaluation period</th></tr>
<tr><th>PART 1</th><th>Decision to move to PART 2</th><th>PART 2</th></tr>
<tr><td colspan="2">Time of implementation</td><td></td><td>Anytime after the end of screening</td><td>Within 6 hours after the end of PART 1</td><td>More than 6 hours have passed since the end of PART 1</td></tr>
<tr><td colspan="2">Confirmation of basic subject information</td><td>○</td><td></td><td></td><td></td></tr>
<tr><td colspan="2">Verify eligibility</td><td>○</td><td></td><td>○</td><td></td></tr>
<tr><td colspan="2">Obtaining Consent</td><td>○</td><td></td><td></td><td></td></tr>
<tr><td colspan="2">MDS-UPDRS</td><td>○</td><td></td><td></td><td></td></tr>
<tr><td colspan="2">Cognitive Function Assessment （MMSE, FAB, MoCA-J）</td><td>○</td><td></td><td></td><td></td></tr>
<tr><td colspan="2">Quality of Life Assessment (PDQ-39)</td><td>○</td><td></td><td></td><td></td></tr>
<tr><td colspan="2">Evaluation of Footfall (FOG-Q)</td><td>○</td><td></td><td></td><td></td></tr>
<tr><td colspan="2">Sham stimulation / SMA stimulation</td><td></td><td>○</td><td></td><td></td></tr>
<tr><td rowspan="5">After Sham stimulation / After SMA stimulation</td><td>Walking distance in 30 seconds</td><td></td><td>○</td><td></td><td></td></tr>
<tr><td>10m Natural Walking Time and Number of Steps</td><td></td><td>○</td><td></td><td></td></tr>
<tr><td>Timed Up and Go test</td><td></td><td>○</td><td></td><td></td></tr>
<tr><td>360° rotation test</td><td></td><td>○</td><td></td><td></td></tr>
<tr><td>Video recording of walking</td><td></td><td>○</td><td></td><td></td></tr>
</table>

	conditions				
Sham stimulation / M1 stimulation					○
After Sham stimulation / After M1 stimulation	Walking distance in 30 seconds				○
	10m Natural Walking Time and Number of Steps				○
	Timed Up and Go test				○
	360° rotation test				○
	Video recording of walking conditions				○
Adverse Events and Defects			○	○	○

*Within 3 months after the end of screening (even if you are readmitted to the hospital after being discharged from the hospital)

12.2. Observation and Inspection Items

(1) Basic information of research subjects: age, gender, history of treatment of diseases
(2) MDS-UPDRS
(3) Cognitive function assessment: MMSE, FAB, MoCA-J
(4) QOL evaluation: PDQ-39
(5) Evaluation of gait questionnaire (FOG-Q)
(6) Evaluation of walking ability: walking distance in 30 seconds, 10m natural walking time and number of steps, Timed Up and Go test
(7) Video recording of walking conditions

12.3. Observation and Inspection Methods

(1) Basic information of study subjects: age, gender, PD treatment history→ excerpts from medical records
(2) Examination by a neurologist → MDS-UPDRS
(3) Evaluation of cognitive function: MMSE, FAB, MoCA-J→ evaluation by a neurologist
(4) QOL evaluation: Based on the PDQ-39 → questionnaire
(5) Evaluation of the freezing of gait questionnaire (FOG-Q) → questionnaire
(6) Evaluation of walking ability: walking distance in 30 seconds, 10m natural walking time and number of steps, Timed Up and Go test, 360° rotation test → conducted in the rehabilitation room
(7) Video recording of the walking state → conducted by the subject in the rehabilitation room.

13. Evaluation items

13.1. Efficacy endpoints

13.1.1. Primary endpoint (primary endpoint)

<PART1>

Comparison of 30-second walking distance between SMA and sham stimulation

<PART2>

Comparison of 30-second walking distance between M1 and sham stimulation

[Rationale for setting]

As a basis for setting the primary endpoint, patients with sloping feet may have a decrease in walking speed, but in severe cases, they may not be able to walk at all when walking, and they may not be able to achieve almost any walking distance. Even in such a case, if the walking distance is taken as the

primary endpoint, it can be left as data. On the other hand, the 10-m walking time set as a secondary endpoint may not be available for such severe cases, and the patient may have to be excluded. Therefore, walking distance was set as the primary endpoint.

13.1.2. Secondary endpoint (secondary endpoint)

10m Natural Walking Time and Number of Steps
Timed Up and Go test
360° rotation test

13.1.3. Exploratory endpoints

Objective evaluation by videography of walking status

13.2. Safety endpoints

Occurrence of adverse events and defects

14. Handling in the event of an outbreak of illness

14.1. Diseases, etc.

14.1.1. Definition of Disease, etc.

"Disease" refers to illness, disability, death, or infectious disease suspected to be caused by the conduct of clinical research, as well as abnormalities in laboratory values and various symptoms.

14.2. Predicted diseases, etc.

Study subjects may experience discomfort such as tingling, itching, headache, or burning sensation on the scalp where the irritation is being applied。 In addition, there is a possibility of bruises and fractures due to falls during gait evaluation.

14.3. Unpredictable diseases, etc.

Diseases other than those specified in section "14.2. Predicted Diseases".

14.4. Determination of severity

In the event that a medical device malfunctions are found to be caused by a malfunction that may cause the following diseases, etc., the following diseases must be reported to the administrator of the medical institution within 30 days from the date of learning of the defect, and the report must be reported to the Accredited Clinical Research Review Board described in the implementation plan.

(1) death
(2) Diseases that may lead to death
(3) Diseases that require hospitalization at a medical institution or extension of the period of hospitalization for treatment
(4) obstacle
(5) Diseases that may lead to disability
(6) (3) ~ (5) and diseases that are serious according to death or diseases that may lead to death, etc.
(7) Congenital diseases or abnormalities in later generations

14.5. Responding to research subjects in the event of an outbreak of a serious illness

In the event that a disease is recognized, the investigator shall immediately take appropriate measures.

In addition, in preparation for compensation liability in the event of health damage to the research subjects in connection with the implementation of this study, this study will be enrolled in clinical research insurance. In the unlikely event that serious health damage (death, permanent disability grade 1 or 2)

occurs due to participation in this study, compensation benefits can be received from the relevant insurance.

14.6 Reporting of illnesses, etc.

In the event that the investigator learns of the occurrence of a disease during the conduct of this study, the investigator shall promptly report to the administrator of the medical institution and report to the Accredited Clinical Research Review Board.

When the Accredited Clinical Research Review Board expresses an opinion on a report of a disease, the principal investigator shall respect the opinion and take necessary measures.

In addition, the report to the administrator of the implementing medical institution and the Accredited Clinical Research Review Board shall be made within the following period.

(1) Outbreaks of any of the following diseases, etc., that are suspected to be caused by the conduct of specific clinical research using unapproved or off-label drugs, etc., and cannot be predicted. 7 days
  (a) Death
  (b) Diseases that may lead to death, etc.

(2) Outbreaks of any of the following diseases, etc., that are suspected to be caused by the conduct of specific clinical research using unapproved or off-label drugs (excluding those listed in (1)): 15 days
  (a) Death
  (b) Diseases that may lead to death, etc.

(3) Outbreaks of any of the following diseases, etc., that are suspected to be caused by the conduct of specific clinical research using unapproved or off-label drugs, etc., and cannot be predicted: 15 days
  (a) Diseases that require hospitalization at a medical institution or extension of the period of hospitalization for treatment
  (b) obstacle
  (c) Diseases that may lead to disability
  (d) Diseases that are as serious as (a) to (c) and death or diseases that may lead to death, etc.
  (e) Congenital diseases or abnormalities in later generations

(4) Matters other than (1) ~ (3) above shall be made at the time of periodic reporting (every year from the date of submission of the implementation plan, within two months after the expiration of the relevant period).

14.7. Remedies

14.7.1. Delivery of salvage drugs, methods of treatment

If the study subject complains of discomfort such as itching, headache, or burning, the stimulation should be stopped immediately.

14.7.2. Emergency treatment such as acute exacerbation

Immediately stop stimulation and immediately take appropriate measures.

15. Statistical matters

15.1. Target number of cases and rationale for setting

Target number of cases: 20

Because this study is an exploratory trial, there is not enough information to calculate the target number of patients based on statistical power. Therefore, from the number of patients hospitalized for the disease (about 500 patients) per year at our hospital, we estimate the number of patients who meet the inclusion criteria and who can consent to participate in the study (about 1 case per month), and the number of patients who can be conducted within the study period (2 years and 6 months) is 20 patients.

15.2. Population to be analyzed

In principle, the target population for efficacy analysis of this study is the target population for

Intention-to-treat (ITT) analysis , which covers all cases, but the following subjects are excluded from the study population.

1) Those who do not meet the inclusion criteria

2) A person who has never taken measurements with a device.

The target population, including subjects who have undergone tDCS stimulation at least once, will be analyzed as the target population for safety analysis.

15.3. Aggregation and Analysis Methods

The background of the study subjects is calculated and aggregated by calculating the mean value and standard deviation for continuous variables, and the frequency and proportion for categorical variables. If the continuous variable clearly does not follow a normal distribution, transform the variable appropriately, such as a logarithmic transformation, and summarize it by mean and standard deviation, or use the median and interquartile ranges as descriptive statistics.

The primary endpoint, the difference in walking distance between 30 seconds after tDCS stimulation and sham stimulation, will be assessed using a single-sample t-test or a Wilcoxon signed rank test.

For the difference in secondary endpoints, 10 m natural walking time and number of steps, Timed Up and Go test, and 360°rotation test after tDCS stimulation and sham stimulation.Evaluate with a one-sample t-test or a Wilcoxon signed rank test.

together with

Video recording of the gait state is performed to objectively analyze the change in the gait state from the perspective of a specialist (neurologist, rehabilitation doctor) when improvement is obtained.

For safety, frequency and proportion (for all subjects) for each adverse event will be evaluated.

15.4. Procedures for handling missing, rejected, and abnormal data

Cases in which the tests and data collection described in this research protocol could not be performed for any reason will be omitted or rejected, and in principle, the missing values will not be supplemented. In addition, for outliers such as outliers, all data are used in the analysis in principle, but in the case of exclusion, the reason for exclusion is recorded. The handling of missing values and abnormal data is described in the statistical analysis plan.

15.5. Procedure for modifying the original statistical analysis plan

If there is a change from the original statistical analysis plan, the research protocol or statistical analysis plan will be revised and explained in the summary report of the clinical study.

15.6. Interim analysis and early discontinuation of the study

No interim analysis was performed in this study.

15.7.Other, Exploratory Analysis

Depending on the results of the study, an exploratory analysis such as a subgroup analysis may be performed. The objectives, items, methods, and results of the analysis are described in the final report.

16. Viewing of original documents, etc.

The Principal Investigator and the Conducting Medical Institution shall make all clinical research-related records, including source materials, directly available for inspection during monitoring and auditing related to clinical research and investigations by accredited clinical research review boards and regulatory authorities.

17. Quality Control & Quality Assurance

17.1. Monitoring & Auditing

17.1.1. monitoring

Implementation Structure

The monitoring of this study will be carried out by a person belonging to the Clinical Research and Clinical Trial Center of Juntendo University Hospital, Juntendo University School of Medicine, designated by the principal investigator in accordance with the monitor designation form.

Contact: 〒113-8431 3-1-3 Hongo, Bunkyo-ku, Tokyo
TEL (direct) 03-381 4-5672 (ext.) 3832

Monitoring Methods

The monitoring of this study will be carried out by central monitoring. The monitor will prepare a monitoring report and submit it to the principal investigator within one month after the monitoring is conducted.

〇Facilities subject to monitoring

Juntendo University School of Medicine Juntendo Hospital Neurosurgery
Juntendo University School of Medicine Juntendo Hospital Department of Neurology

Timing of monitoring

The study will be monitored once at the end of the study

Items to check for monitoring

The monitor checks the following:

① Patient enrollment status: Number of registrations, cumulative
② Eligibility: Patients who may be ineligible
③ Pre-treatment background factors (basic information of study subjects)
④ Between/after study treatment, discontinuation, and reason
⑤ Whether or not there is a non-conformity, and what it is
⑥ Whether or not there is a malfunction of the equipment, such as illness, etc., its contents, and the status of response
(7) Other issues related to the progress of testing and safety

17.1.2. audit

Implementation Structure

The audit of this study will be conducted by a person designated by the principal investigator with an auditor designation letter at the appointment of the Director of the Clinical Research Compliance and Governance Promotion Office, Clinical Research and Clinical Trials Center, Juntendo University Hospital.

Contact: 〒113-8431 3-1-3 Hongo, Bunkyo-ku, Tokyo
TEL (direct) 03-3814-5672 (ext. 3832)

How to Conduct an Audit

(1) The audit of this research will be conducted by visiting the facility.

〇Facilities subject to audit

・Department of Neurosurgery, Juntendo University School of Medicine
・Department of Neurology, Juntendo University School of Medicine

(2) Direct inspection (SDV) shall be carried out in accordance with the procedures of the medical institution.

When to conduct an audit

Audits of this research will be conducted at the end of this research and when other needs arise. The auditor shall submit the audit report to the principal investigator within one month after the completion of the audit.

○ Audits to be conducted at the end of the research

After the monitoring of this study is completed, an audit will be conducted.

○ Audits to be conducted when the need arises

The audits to be conducted when necessary for this research are as follows.

(A) When a disease report is submitted to the Minister of Health, Labour and Welfare

(B) When serious non-conformity* with the law, enforcement regulations, or research plan is discovered.

(C) In addition, when the principal investigator deems it necessary.

* Serious non-conformity is defined as one that affects the human rights, safety, progress of research, and reliability of results (e.g., non-compliance with inclusion/exclusion criteria, discontinuation criteria, etc.). In order to avoid immediate danger to the subject, those who did not comply with the research plan for other medical compelling reasons are not included.

<u>Audit Checks</u>

The audit of this study will confirm the following items.

- ･Documents related to the procedures of the Clinical Research Review Board
- ･Consent form obtained from the research subject, withdrawal of consent form
- ･List of research subject identification codes
- ･Storage and management status of personal information
- ･Confirmation of case eligibility
- ･Status of medical examinations and examinations
- \- Number of cases of discontinuation of research and reason for discontinuation
- ･ Presence of equipment malfunctions, such as diseases, contents, and response status
- \- Presence, content, and status of non-conformity (including serious non-conformance) with the Clinical Research Act or research plan
- ･Monitoring results and storage status of monitoring reports
- ･Other matters required by the principal investigator

17.2. Data Management

In this study, we use CRF and REDCap as a management tool for data management. After the data is fixed, Yanagisawa, who is in charge of statistical analysis, performs statistical analysis.

18. Ethical Considerations

18.1. Rules to be observed

All persons involved in this research shall carefully read and understand the contents of the "Declaration of Helsinki of the World Medical Association," the "Ethical Guidelines for Medical Research on Human Subjects," and the "Clinical Trials Act," which all medical research involving human subjects should comply with.

18.2. Protection of personal information and privacy of research subjects

Persons involved in research shall comply with applicable laws and ordinances regarding the protection of personal information of research subjects. In addition, the relevant parties shall make every effort to protect the personal information and privacy of the research subjects, and shall not divulge personal information obtained in the course of conducting this research without a justifiable reason. The same shall apply even after the person concerned has retired from the position.

When handling personal information related to research conduct, each participating facility (personal information manager, etc.) shall manage it with a research ID that is not related to personal information, and give due consideration to the confidentiality of the research subject. The correspondence table prepared is strictly managed by the principal investigator in a locked locker in the neurosurgery laboratory.

19. Explanation and method of obtaining consent from the subject of clinical research

19.1. Summary of the burden and anticipated risks and benefits to the study subjects

Participation in this study will not create any new financial burden on the study subjects.

There is no projected direct benefit for the study subjects to participate in the study, and the same applies to the disadvantages.

19.2. Projected Profits

Although it is unclear whether the study participants will benefit from participating in this study, the results of this study may contribute to future medical advances by improving the improvement of refractory feet and applying to rehabilitation, for which there is currently no effective treatment.

There are no new costs associated with the treatments being tested in this study. In addition, since all medical expenses, including drug costs, during the study period of the study subjects are paid by the patient's insurance and the patient's own expenses, there is no special medical or economic benefit that can be obtained by the study subjects by participating in the study.

19.3. Anticipated Dangers and Disadvantages and Measures to Minimize Them

Transcranial direct current electrical stimulation is based on the recommendation from the committee of the Japanese Society of Clinical Neurophysiology that stimulation at an intensity of 3 mA for up to 30 minutes is safe, and stimulation is performed within this range. In fact, the stimulator used in this study is actually a commercially available device, and the maximum output is 2.0 mA, and it is designed so that stimulation cannot be used for more than 20 minutes per day.

However, as an adverse event, it is expected to cause some tingling in the scalp. If the stimulus is unpleasant, respond by reducing the intensity of the stimulus. In addition, if the subject complains of discomfort such as itching, headache, or burning, the stimulation should be stopped immediately.

19.4. Steps to obtain consent

The principal investigator and co-investigator shall fully explain the contents of the study before participating in this clinical study based on the explanation and consent documents separately stipulated for the research subjects.

The explanation and consent documents shall be prepared by the principal investigator and used after obtaining the approval of the Accredited Clinical Research Review Board. If it is to be revised, it must be applied to the Accredited Clinical Research Review Board again and used after obtaining approval.

After giving the investigator sufficient time to think about whether or not to participate in the clinical study, the principal investigator and the co-investigator will obtain the consent of the investigator to participate in the study of the student's free will in writing (a consent document to be specified separately).

19.5. Contents of the Consent Briefing Document

The items to be included in the consent document shall be as follows.

1) The name of the clinical study, the fact that the director of the research institution has received permission to conduct the clinical study, and that the implementation plan has been submitted to the Minister of Health, Labour and Welfare
2) The name of the research institute and the name and title of the principal investigator (in the case of conducting research jointly with another research institute, the name and title of the principal investigator, the name of the joint research institute, and the name and title of the principal investigator of the joint research institute are included.) ）

3) Purpose and Significance of Clinical Research
4) Methods of clinical research (including the purpose of use of samples and information obtained from research subjects)) and period
5) Reasons for being selected as a research subject
6) Burdens and anticipated risks and benefits to the study subjects
7) The fact that even if you agree to the conduct or continuation of clinical research, you can withdraw it at any time (if it may be difficult to take measures in accordance with the contents of the withdrawal from the research subject, etc., that fact and the reason for it)
8) The fact that the research subject will not be treated unfavorably by not agreeing to the clinical research being conducted or continued, or by withdrawing the consent.
9) Methods of Disclosure of Information on Clinical Research
10) At the request of the research subject or his/her representative, the research protocol and materials related to the method of clinical research can be obtained or viewed to the extent that it does not interfere with the protection of personal information, etc. of other research subjects, etc., and the method of obtaining or viewing such materials as long as it does not interfere with the protection of personal information, etc. of other research subjects, etc. and the originality of the research.
11) Handling of personal information, etc. (including the method of anonymization, and the fact that anonymized or non-identifiable processed information is created) ）
12) Methods of storage and disposal of samples and information
13) Conflicts of interest related to research by researchers, etc., such as sources of funding for clinical research, conflicts of interest related to research at research institutions and individual profits, etc.
14) Responding to complaints and inquiries from research subjects and related parties
15) If there is a financial burden or gratuity on the research subject, etc., that fact and the details thereof
16) In the case of clinical research involving medical treatment that exceeds normal medical care, matters related to other treatment methods, etc.
17) In the case of research that involves medical treatment beyond normal medical care, measures to be taken regarding the provision of medical care to the research subject after the research is conducted.
18) If there is a possibility that important knowledge about the health of the research subject, genetic characteristics that can be passed on to descendants, etc. may be obtained as a result of the clinical study, the results of the research related to the research subject (including incidental findings) will be distributed. Handling of
19) In the case of invasive clinical research, whether or not there is compensation for the health damage caused by the research and the details thereof.
20) If there is a possibility that samples and information obtained from research subjects will be used for future research that is not specified at the time of obtaining consent from research subjects, etc., or that may be provided to other research institutions, that fact and the content envisioned at the time of receiving consent.
21) Invasiveness (except for minor invasions) In the case of clinical research involving intervention, the person engaged in monitoring, the person engaged in the audit, and the Accredited Clinical Research Review Board will inspect the samples and information related to the research subject to the extent necessary, on the premise that the confidentiality of the research subject will be preserved.

20. Records (including data) Handling and storage of

20.1. Whether or not samples and information are provided to other organizations

20.1.1 Whether or not samples and information are provided to other organizations

□ Yes
☑ No

20.1.2 Methods of storage and disposal of samples and information

This research does not apply because it does not provide samples or information to other institutions.

20.2. Storage of samples and information related to research

The principal investigator shall keep documents related to the conduct of the research (such as a copy of the application documents, a notification document from the hospital director, a copy of the application form and report, a list of research subject identification codes, a consent form, a copy of the case report form, and other documents or records necessary to guarantee the reliability of the data) and retain them for five years after the end of the study.

When disposed of, it shall be disposed of in an anonymized state so that individuals cannot be identified.

20.3. Secondary Use of Samples and Information Obtained from Research Subjects

The data obtained in this study may be used for secondary purposes in a form that is not linked to personally identifiable information, only if it is approved after review by the Accredited Clinical Research Review Board.

21. Results of research on research subjects (including accidental findings) when important findings were obtained regarding the health of the research subjects, genetic characteristics that can be passed on to offspring, etc. Disclosure of

This study is not applicable because it is not a study on the health of the research subjects or the genetic characteristics that can be passed on to their descendants.

22. Payment of money and compensation for the conduct of clinical research

22.1. Whether or not you have insurance and what it is

☑ Subscribe

□ Don't subscribe

If you subscribe to <, the contents >

Clinical Research Liability Insurance

22.2. Compensation and reparation for health damage

In preparation for the liability for compensation in the event of health damage to the research subjects in connection with the implementation of this study, this clinical study will be enrolled in clinical research insurance. In the unlikely event that serious health damage (death, permanent disability grade 1 or 2) occurs due to participation in this study, the researcher can receive compensation benefits from the insurance to which the researcher is enrolled.

In addition, after the completion of the research on each research subject, efforts will be made to ensure that the best medical care (preliminary, diagnosis and treatment) obtained from the results of the study can be received.

22.3. Projected medical expenses (burden on the study subject)

There is no cost burden for the study subjects by participating in this study. Only burdens that fall under general insurance medical treatment are incurred.

22.4. Payment of money to research subjects, subsidy of medical expenses

There will be no payment of money or subsidies for medical expenses to the study subjects by participating in this study.

23. Publication of information on clinical research

23.1. Register for research

Information on research is registered in the Japan Registry of Clinical Trials (RCT, URL: https://jrct.niph.go.jp/), a public database established by the Ministry of Health, Labour and Welfare

23.2. Updates on research information

j The information registered in the RCT will be updated as appropriate.

23.3. Attribution of research results and publication of results

The results of this study will be presented at the Parkinson's Disease and Movement Disorders Society and published as papers in academic journals specializing in the field of movement disorders. In any case, only the results that have been statistically processed will be disclosed, and no personal information of the research subjects will be disclosed.

The principal investigator shall prepare a primary endpoint report within one year from the date of the end of the primary endpoint data collection period, and a summary of the summary report and summary report within one year from the date of the end of the collection period of all data, and publish it to the J RCT within one month from the date of hearing the opinion of the Accredited Clinical Research Review Board.

24. Matters necessary for the proper conduct of clinical research

24.1. Provision of research funds by pharmaceutical manufacturers, distributors, etc. for this clinical research

□ Yes

☑ No

If there is a <, its contents>

24.2. Sources of research funding

This research uses a Grants-in-Aid for Scientific Research provided by the Japan Society for the Promotion of Science, Japan.

Name of Research Category: Scientific Research (C) (General)

Research ID number: 19K09465

Project Title: Neuromodulation by Transcranial Direct Current Electrical Stimulation for Limp Feet in Parkinson's Disease

24.3. Conflict of Interest

In this research, we will use a research grant provided by the Grant-in-Aid for Scientific Research of the Japan Society for the Promotion of Japan Science. Regarding the management of conflicts of interest, the principal investigator submits the conflict of interest management standards and the conflict of interest management plan to the Accredited Clinical Research Review Board for approval in accordance with the Conflict of Interest Management Guidance for Clinical Research in the Clinical Trials Act.

25. Handling of Case Report Forms (CRFs)

The Principal Investigator shall provide important documents related to the conduct of the study (case report form (CRF): records regarding the date, time, and place of use of medicines, etc. for each subject of the study, research plan, implementation plan, documents related to explanation and consent to the subjects of this study, summary reports, documents related to review opinions received from the Accredited Clinical Research Review Board, Documents related to monitoring and auditing, contracts for the implementation of this research, documents outlining the drugs used in this research, and other documents necessary for conducting this research) shall be stored at a place designated by the principal investigator of each conducting medical institution until the date when five years have elapsed since the discontinuation or termination of the study, and then personal information shall be disposed of with care. The details of the handling of CRFs are stipulated in the Data Management Manual. The final results of the study belong to Juntendo University.

26. Revision of the research protocol

Collect and review information necessary for the safe conduct of clinical research. In addition, if new safety information is obtained, the research plan and consent briefing will be revised as necessary. If it becomes necessary to revise the research plan or consent document, it will be revised in accordance with the procedures established by the Accredited Clinical Research Review Board.

The record and reason for the revision will be described in the "List of Renewal and Approval History" shown on the cover.

27. Responding to consultations from research subjects and related parties

The consultation desk for this clinical research will be established as follows.

【Consultation Desk】

Principal investigator: Atsushi Umemura, Specially Appointed Professor, Department of Neurosurgery, Juntendo University School of Medicine

〒113-8431 3-1-3 Hongo, Bunkyo-ku, Tokyo Juntendo University School of Medicine Juntendo Hospital Neurosurgery

Phone number: 03-3813-3111 (ext. 71008) E-mail: aumemura@juntendo.ac.jp

28. Measures related to the provision of medical care to research subjects after the conduct of the research

After the study is completed, treatment with regular insurance treatment will be continued.

29. References

1) Zhou C, Zhong X, Yang Y, Yang W, Wang L, Zhang Y, Nie K, Xu J, Huang B: Alterations of regional homogeneity in freezing of gait in Parkinson’s disease. J Neurol Sci 387: 54-59, 2018

2) Katsuya Ogata and Shozo Tobimatsu: Fundamentals and clinical applications of transcranial direct current stimulation (tDCS).Measurement and control 54: 106-113, 2015

3) Hikaru Kirimoto and Hideaki Onishi: Modification of central nervous system excitability using transcranial direct current electrical stimulation and its clinical application.Physical Therapy 44: 166-177, 2017

4) Edwards DJ, Cortes M, Wortman-Jutt S, Putrino D, Bikson M, Thickbroom G, Pascual-Leone A: Transcranial direct current stimulation and sports performance. Front Hum Neurosci 11: 243, 2017

5) Huang L, Deng Y, Zheng X, Liu Y: Transcranial direct current stimulation with halo sport enhances repeated sprint cycling and cognitive performance. Front Hum Neurosci 10: 118, 2019
